# Population Genetic Structure and Connectivity of the European Lobster *Homarus gammarus* in the Adriatic and Mediterranean Seas

**DOI:** 10.3389/fgene.2020.576023

**Published:** 2020-12-07

**Authors:** Mišo Pavičić, Iva Žužul, Sanja Matić-Skoko, Alexandros Triantafyllidis, Fabio Grati, Eric D. H. Durieux, Igor Celić, Tanja Šegvić-Bubić

**Affiliations:** ^1^Institute of Oceanography and Fisheries, Split, Croatia; ^2^School of Biology, Aristotle University of Thessaloniki, Thessaloniki, Greece; ^3^Institute for Biological Resources and Marine Biotechnologies (IRBIM), National Research Council (CNR), Ancona, Italy; ^4^UMR CNRS 6134 Sciences Pour l’Environnement, Università di Corsica Pasquale Paoli, Corte, France; ^5^UMS CNRS 3514 STELLA MARE, Università di Corsica Pasquale Paoli, Biguglia, France; ^6^National Institute of Oceanography and Applied Geophysics – OGS, Trieste, Italy

**Keywords:** gene flow, genetic diversity, population structure, fisheries, microsatellites, mtDNA

## Abstract

Highly selective fishing has the potential to permanently change the characteristics within a population and could drive the decline of genetic diversity. European lobster is an intensively fished crustacean species in the Adriatic Sea which reaches high market value. Since knowledge of population structure and dynamics is important for effective fisheries management, in this study, we used 14 neutral microsatellites loci and partial mitochondrial COI region sequencing to explore population connectivity and genetic structure by comparing samples from the Adriatic Sea and the adjacent basins of the Mediterranean Sea. The obtained results suggest that neutral genetic diversity has not been significantly affected by decrease in population size due to overfishing, habitat degradation and other anthropogenic activities. Global genetic differentiation across all populations was low (*F*_*ST*_ = 0.0062). Populations from the Adriatic Sea were panmictic, while genetic differentiation was found among populations from different Mediterranean basins. Observed gene flow for European lobster suggest that populations in the north eastern Adriatic act as a source for surrounding areas, emphasizing the need to protect these populations by establishing interconnected MPAs that will be beneficial for both fisheries and conservation management.

## Introduction

Genetic connectivity among marine populations is an important driver for ecological evolutionary processes of species and reflects historical and contemporary demographic events, such as climate shifts, environmental features, oceanographic patterns or particular species behaviors (Avise, 2000; Selkoe et al., 2008). Larval dispersal, juvenile or adult stages migration and recruitment success through marine ecosystems shapes connectivity between populations, influencing fisheries management and establishment of marine reserve networks (Shanks et al., 2003; Fogarty and Botsford, 2007). Spatial connectivity of many marine species is a result of the complex interplay of different life-history traits and their environmental change responses, where transport of planktonic larval stages by sea currents may last for several days to weeks before settlement, followed by a sedentary adult phase (Cowen et al., 2007). However, some species with long larval periods show spatially limited dispersal and self-recruitment patterns to natal habitats, despite oceanic currents (Cowen et al., 2006). Thus, prediction of population connectivity in the marine environment presents a challenging task.

Genetic approaches based on gene flow and diversity estimations are highly recommended for effective conservation decisions, since genetic diversity has been one of the most important biodiversity conservation issues (Laikre et al., 2010). On the other hand, intense fishing pressures can negatively impact the genetic diversity of exploited populations through selection and genetic drift (Marty et al., 2015). Therefore, knowledge of stock composition and population dynamics of exploited species is important for sustainable management of fisheries (Ovenden et al., 2015). Fisheries science successfully applies population genetic approaches for this purpose (Cadrin et al., 2014), including determination of fisheries stock structure, population abundance, genetic diversity and evolutionary response to exploitation (Ovenden et al., 2015; Matić-Skoko et al., 2018). Generally, genetic studies on decapod crustaceans and associated management plans in the Mediterranean are scarce. Although, the European lobster (*Homarus gammarus*) is a commercially important species for Croatian small-scale fisheries, the knowledge of its genetic diversity, stock structure and population dynamics in the Adriatic Sea is currently lacking.

The species is distributed in the north-eastern Atlantic Ocean from Morocco (though absent in the Baltic Sea) to Arctic Norway, and in the Mediterranean Sea (Triantafyllidis et al., 2005; Supplementary Figure 1). The European lobster inhabits the continental shelf from the low intertidal to 150 m depth, typically not deeper than 50 m, and favors hard substrates like rock or hard mud (Holthuis, 1991). They are territorial and nocturnal animals inhabiting crevices and holes (Skerritt et al., 2015). Females mature sexually between the ages of 5 and 8 with a carapace length of 80 to 85 mm (Holthuis, 1991). Fertilized eggs develop for 10 to 12 months on the female pleopods and the planktonic larvae spend 15 to 35 days drifting where water temperature plays significant role in development (Browne et al., 2009). This is a highly prized marine species targeted by small-scale fishers throughout its range. Over the past decade, the total European annual landings varied between around 4500 and 5600 tons (FAO, 2020), mostly originating from the northern European countries. Landings in Mediterranean countries have never reached those levels (Prodöhl et al., 2007). In the past, intensive fishing in Scandinavia and the Mediterranean led to stock depletions and recovery did not occur or was slow (Ellis et al., 2015b). The European lobster is present in the Adriatic Sea, it is more abundant in its northern part and in Croatia it is fished traditionally with baited traps, trammel nets and gillnets targeting fish.

In the last two decades, genetic studies have been carried out intensively, especially in northern European regions where stock enhancement programs were implemented to supplement fisheries production in heavily depleted areas (Jørstad et al., 2005; Triantafyllidis et al., 2005; Ellis et al., 2015a, 2017; Watson et al., 2016; Jenkins et al., 2019). Several genetic distinct clusters of European lobster have been identified across its range of distribution (Triantafyllidis et al., 2005; Ellis et al., 2017). Genetic division was noticed between European lobster populations from the Atlantic Ocean and from the Mediterranean Sea. In the Mediterranean, population structuring was also evident between the central Mediterranean and the Aegean Sea (Jenkins et al., 2019). However, strong conclusions of additional structuring among Mediterranean populations could not be reached because of the limited number of populations sampled across basins, e.g., populations from central (Ionian and Adriatic Sea) and western Mediterranean (Balearic and Alboran Sea) are lacking. In particular, complete genetic structure analysis of European lobster has not been previously conducted for the Adriatic Sea, except for south Adriatic Sea which was represented with one sampling site (Triantafyllidis et al., 2005).

In the present study, the population genetic variation of European lobster was investigated in the Adriatic Sea and the adjacent basins of the Mediterranean Sea using two classes of genetic markers, i.e., 14 neutral microsatellites and a sequence polymorphism of a mitochondrial DNA COI region. Based on sampling coverage, this study aimed to: (i) provide detailed insights into the genetic structure of European lobster in the Adriatic Sea and assess genetic connectivity between the Adriatic and Mediterranean basins; (ii) detect the possible impacts of intense fishing pressure on species genetic diversity and reconstruct its demographic history based on mtDNA and nuclear markers, and (iii) consider potential management implications of present findings for European lobster in the Adriatic Sea.

## Materials and Methods

### Tissue Sampling and DNA Extraction

**FIGURE 1:**
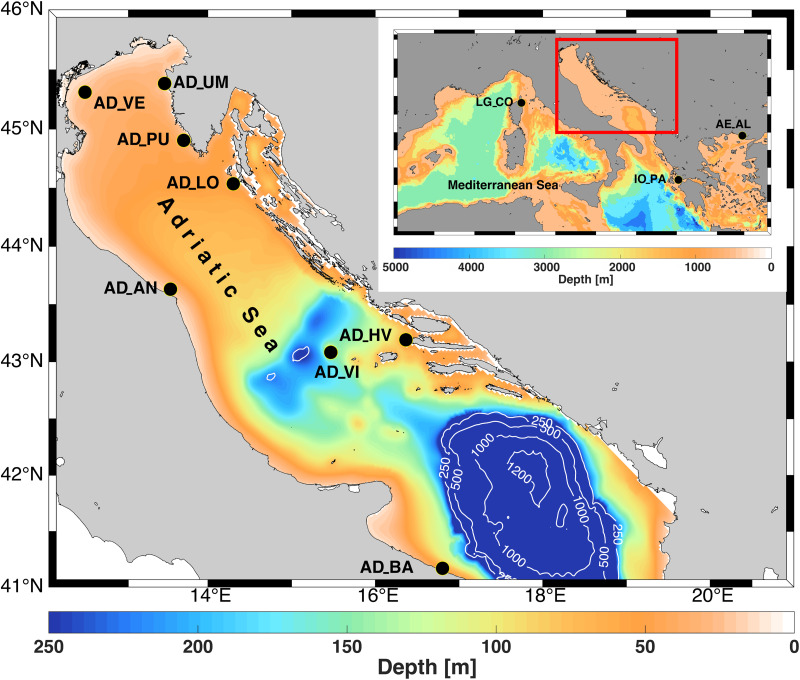
Central and eastern Mediterranean Sea bathymetry with sampling locations of European lobster *Homarus gammarus* where the first two letters of location abbreviations denote the geographic origin of samples (AD, Adriatic Sea; IO, Ionian Sea; AE, Aegean Sea; LG, Ligurian Sea) and the second two letters denote the abbreviations for sampling sites (VE, Venice; UM, Umag; PU, Pula; LO, Lošinj; AN, Ancona; HV, Hvar; VI, Vis; BA, Bari; CO, Corsica; PA, Patras; AL, Alexandroupoli). Information on populations included and regional subdivisions are provided in Table 1. Isobaths indicate depths of 250, 500, 1000, and 1200 m. The colors represent different depths. The figure was prepared in MATLAB 2014a (www.mathworks.com) and GIMP 2.8.16 (www.gimp.org) software.

A total of 331 individuals of European lobster were collected from the central and eastern Mediterranean (Figure 1 and Table 1). The Adriatic Sea was intensively sampled at eight different locations of the north and mid Adriatic on both the eastern and western sides of the basin during 2017 and 2018. Sampling was primarily conducted in collaboration with local commercial fishing vessels. For each captured European lobster, a tissue sample (pleopod clip) was taken, and sex and carapace length (mm) measured. Other samples, in the form of extracted DNA from Greece (2001) and preserved tissues from Corsica (2015–2017) were obtained from collaborators, including information of the approximate fishing region and date of capture. Sample sizes ranged from 4 to 48 per location. For samples from the Adriatic Sea of known length, a carapace length of 87 mm was used as a boundary to discriminate adults from juveniles (Phillips, 2013). Total genomic DNA was extracted from individual pleopod tissues by proteinase K digestion and later was applied standard phenol-chloroform extraction protocol. DNA quality and quantity was checked with the help of **s**pectrophotometer (IMPLEN N50, Germany).

**TABLE 1:** Sampling information, codes and the number of individuals of European lobster *Homarus gammarus* genetically tested with 14 putative neutral microsatellites (SSR) and mtDNA COI marker.

Site	Country (GSA)	Pop ID divided by age	No. samples		Sampling year	Latitude	Longitude
			SSR	mtDNA			
Venice	Italy (GSA 17)	AD_VE_A	20	–	2018	45.311057	12.531770
Umag	Croatia (GSA 17)	AD_UM_A	48	7	2017–2018	45.385039	13.4825
		AD_UM_J	8	–			
Pula	Croatia (GSA 17)	AD_PU_A	48	3	2017–2018	44.904897	13.710469
		AD_PU_J	24	–			
Lošinj	Croatia (GSA 17)	AD_LO_A	34	8	2017–2018	44.536533	14.303519
Ancona	Italy (GSA 17)	AD_AN_A	14	6	2018	43.631994	13.553164
		AD_AN_J	16	–			
Hvar	Croatia (GSA 17)	AD_HV_A	39	11	2017–2018	43.191894	16.366064
Vis	Croatia (GSA 17)	AD_VI_A	28	14	2017–2018	43.083017	15.468786
Bari	Italy (GSA 18)	AD_BA_A	–	3	2017	41.168583	16.803222
Corsica	France (GSA 8)	LG_CO_A	28	5	2015–2017	42.6373	9.475144
Patras	Greece (GSA 20)	IO_PA_A	4	2	2001	38.291290	21.127216
Alexandroupoli	Greece (GSA 22)	AE_AL_A	20	4	2001	40.816339	25.869478
**Overall**			331	63			

*The first two letters of pop ID abbreviations denote the geographic origin of samples (AD, Adriatic Sea; IO, Ionian Sea; AE, Aegean Sea; LG, Ligurian Sea), and the second two letters denote the abbreviations for sampling sites, while the last letter characterizes the ontogenetic state of individuals (A, adult; J, juvenile).*

### Microsatellite Genotyping and Analyses

DNA samples were genotyped with 15 microsatellite markers designed for European lobster, following the protocol outlined in Ellis et al. (2015a) where 12 microsatellites from André and Knutsen (2010) and 3 microsatellites from Ellis et al. (2015a) were combined in three multiplex reactions (Table 1 and Supplementary Table 1). On an Eppendorf Mastercycler Nexus Gx2 thermal cycler, microsatellites were amplified in 10 μL reactions with a 10 ng DNA template using the Multiplex PCR kit (Qiagen, Germany). Final concentrations of 0.2 μM were uniformly set for all primers. For all multiplex PCRs, the same protocol was used with the following steps: initial denaturation at 95°C for 5 min, followed by 26 cycles of denaturation at 95°C for 30 s, annealing at 60°C for 90 s, and elongation at 72°C for 30 s; and the final elongation at 60°C for 30 min. Genotyping was performed on an ABI 3730 (Applied Biosystems) DNA sequencer with an internal size standard 500 LIZ dye Size Standard (Applied Biosystems). Genemapper v.3.5 (Applied Biosystems) was used to call allele sizes.

### Genetic Diversity

The presence and frequency of null alleles for each locus was checked by MICROCHECKER v.2.2.3 (Van Oosterhout et al., 2004) and FREENA (Chapuis and Estoup, 2007). The fixation index (*F*_*IS*_), linkage disequilibrium (LD), departures from Hardy-Weinberg equilibrium (HWE), observed and expected heterozygosity (*H*e, *H*o) were assessed using GENEPOP 4.0 (Raymond and Rousset, 1995; Rousset, 2008). The average number of alleles per locus (A) and effective number of alleles across loci (Ae) were estimated in POPGENE 1.32 (Yeh et al., 1999). Allelic richness (Ar) and differences in allelic richness and heterozygosity among populations were calculated and tested in FSTAT 2.9.3 (Goudet, 2002). Whenever needed, the sequential Bonferroni correction was used for multiple testing (Rice, 1989).

The relationship between fitness and heterozygosity can be investigated by measuring genetic diversity. Four heterozygosity measures for each individual with known carapace length and sex were estimated as the proportions of heterozygous genotypes with the function GENHET.R (Coulon, 2010) in R (R Core Team, 2017). To investigate whether sex or fitness components of individuals are correlated with their level of heterozygosity, linear regressions were employed in STATISTICA (see Supplementary Material for details).

### Genetic Differentiation and Population Structuring

The level of gene flow among sampling locations was estimated with pair-wise and global *F*_*ST*_ values over 14 loci in ARLEQUIN v.3.5, using 10,000 permutations for testing statistical significance. The Dest measure based on allele identities (Jost, 2008) was calculated in GENODIVE (Meirmans and Van Tienderen, 2004). To assess the contribution of null alleles in the dataset and its impact on the level of population differentiation, FreeNA was applied to compute the *F*_*ST*_ statistic, both with exclusion and inclusion of the Excluding Null Alleles correction method. Using 50,000 replicates over loci, the bootstrap 95% confidence intervals (CI) for the global *F*_*ST*_ values were estimated.

Bayesian-clustering program STRUCTURE 2.3 (Pritchard et al., 2000) and the Discriminant Analysis of Principal Components (DAPC) method implemented in the Adegenet package (Jombart, 2008) for R (R Core Team, 2017) were used for genetic structure analysis of the sampled populations. Set-up parameters for STRUCTURE included usage of the admixture ancestry model with correlated allele frequencies, a burn-in period of 50^3^ iterations and 50^4^ MCMC (Markov chain Monte Carlo) steps. *K*-value ranged from 1 to the maximal number of sampled groups, including 20 replicates for each value. Using Structure Harvester 0.6.93 (Earl and von Holdt, 2012), the most likely number of clusters was assessed with ln *P*(*D*) and delta *K*-values and visualized using CLUMPP (Jakobsson and Rosenberg, 2007) and DISTRUCT (Rosenberg, 2004). The DAPC was executed by the function dapc with sampling location used as a prior. Retained number of Principal Components (PCs) was optimized executing the function xvalDapc (Jombart, 2008).

Isolation by distance (IBD) analysis was tested with the package *Adegenet* and the *mantel.randtest* function in R using 1000 permutations. To explore pattern of IBD, described as a continuous or distant patchy cline of genetic differentiation, two-dimensional kernel density estimation (function kde2d) was applied to measure local densities of distances and function image for plotting in the package MASS (Venables and Ripley, 2002).

### Test of Demographic Changes, Effective Population Size and Migration Rate

BOTTLENECK 1.2.02 (Piry et al., 1999) was used in order to detect recent declines in effective population sizes (*N*e) over a relatively short period (several *N*e generations). With 20,000 replications, two-phase model (TPM) with 90% single-step mutations and 10% variance among multiple steps was tested for populations with a sample size over 20 individuals. The probability of significant heterozygote excess was tested using a one-tailed Wilcoxon signed-rank test, as recommend by Piry et al. (1999). To further investigate the reduction of population size, the *M* ratio test was conducted in MPVal and the critical value of Mc was estimated by CriticalM software for each population (Garza and Williamson, 2001). The M ratio depends on the total number of alleles (*k*) and overall range in allele size (*r*) at a given locus. In recently reduced populations, *M*-ratios is expected to be smaller than in populations under mutation-drift equilibrium and, as such, may be persevered for hundreds of generations (Garza and Williamson, 2001) in contrast to the heterozygosity excess outcomes. The *M*-ratio empirical values were compared to the bottleneck threshold of 0.68 (Garza and Williamson, 2001) and to the simulated equilibrium distribution formed on the two-phase mutation model. With simulation of 10,000 replicates, Mc was calculated engaging the mean size of non-stepwise mutations (Δ_*g*_) = 3.1, and the proportion of stepwise mutations (*p*_*s*_) = 78% (Peery et al., 2012). Three different values (0.5, 1, 4) were set for theta (Θ = 4*N*eμ) that correspond to a pre-bottleneck effective population size of 250, 500, and 2000, respectively.

Contemporary gene flow based on few generations (m*c*) and historical gene flow that covers much longer period of time (∼ 4 *N*e generations in the past, m*h*) were estimated among populations by BayesAss1.3 (Wilson and Rannala, 2003) and Migrate (Beerli and Felsenstein, 2001; Beerli, 2006). For BayesAss, several analyses with different starting seeds were performed and each Markov chain Monte Carlo run involved 3 × 10^6^ iterations and discarding of the first 10^6^ iterations as burn-in. The delta values DA, DF, and DM were set to 0.7, 0.6, and 0.5, respectively. Log-probability of each run was analyzed with TRACER v.171 (Rambaut et al., 2018) and the best model fit was determined based on Bayesian deviance measure (Spiegelhalter et al., 2002). Migrate v 3.7.2 (Beerli, 2008) and application of Bayesian inference was used to estimate gene flow, i.e., mutation-scaled rates of migration (*M* = *m*_*h*_/*μ*; *m*_*h*_ is the historical migration rate; *μ* is the mutation rate per generation) and mutation-scaled effective population size (Θ = 4*N*eμ; *N*e is historical effective population size). The Brownian motion mutation model and *F*_*ST*_ calculations as starting parameter values were used with constant mutation rates for all loci. Uniform prior distribution was applied to estimate Θ (range = 0–50, mean = 25) and *M* (range = 0–50, mean = 25). A long chain was set to visit 1,000,000 parameter values with a burn-in of 50,000 iterations for each locus and to record 20,000 genealogies with a sampling increment of 50. To improve the MCMC searches, static heating scheme at four temperatures (1, 1.5, 3, and 6) was applied.

NeEstimator V2 (Do et al., 2014) program that implements the single sample linkage disequilibrium method was used to estimate contemporary effective population size for populations with a sample size over 20 individuals. Alleles with frequencies bellow 0.02 were removed from the analysis, balancing between precision and potential bias of highly polymorphic microsatellites loci. In addition, the likelihood method implemented in AgeStructure (Wang et al., 2010) was applied to estimate the generation interval and effective size of European lobster of known length size and sex (Adriatic samples). Sex, age, and multilocus genotype information of 159 males and 107 females were provided for *N*e estimation of an age structured lobster population with overlapping generations. Following Uglem et al. (2005), age calculations based on carapace length were performed.

### Mitochondrial DNA (mtDNA) Sequencing, Genetic Diversity and Population Expansion

A part of the mtDNA-encoded cytochrome *c* oxidase subunit 1 (*COI*) gene was amplified for the reduced dataset (see Table 1 for population codes) with the LCO and HCO primer pair (Folmer et al., 1994), following the PCR protocol reported in Pavičić et al. (2020). Product purification and sequencing were performed by Macrogen Inc (Amsterdam, Netherlands) on an ABI 3730 automatic sequencer. Difficulties to secure fresh samples were experienced. So, part of the samples that were successfully genotyped for microsatellite markers had to be excluded from mtDNA analysis due to the poor product amplification. Sequence alignment was performed in Mega7 using the ClustalW tool (Kumar et al., 2016). Molecular diversity was measured using Dnasp 5.19 (Librado and Rozas, 2009) calculating the number of haplotypes (H), polymorphic sites (S), haplotype and nucleotide diversity. The median-joining haplotype network was constructed in PopART^1^.

Demographic history changes were assessed by Tajima’s D (Tajima, 1989) and Fu’s FST (Fu, 1997) tests in Arlequin 3.5.1.2 (Excoffier and Lischer, 2010). Test values indicate a recent population bottleneck or population expansion. The mismatch distribution simulated in Arlequin 3.5.1.2 was analyzed using the sudden expansion model. Model fit was statistically tested using the sum of square deviations (SSD) to reject the hypothesis of demographic expansion. Using Arlequin (Rogers and Harpending, 1992), the time since population expansion (*t*) was estimated as *t* = τ/2*u*, where τ is the mutational time scale. The mutation rate per locus per generation (2*u*) was calculated by multiplying the length of sequence × mutation rate × generation time. In the absence of the calibrated mutation rate of the mtDNA of European lobster, the mutation rates from 1.66 to 2.33% per million years of *Sesarma* COI gene were tested (Schubart et al., 1998). The lower CI boundary of 9.81 years was used as the generation time for European lobster.

## Results

### Genetic Diversity

A total of 331 individuals of European lobster were genotyped at 15 microsatellite loci (Table 1, Figure 1, and Supplementary Table 1). The amount of missing data per locus and population ranged from 0 to 3.5%, with an average of 0.04%. MICROCHECKER detected a high frequency (>10%) of null alleles for the locus HGA8 and accordingly, that locus was removed from further analyses. The loci HGC120 and HGC129 showed the presence of null alleles at low frequencies (<4%) and therefore were kept on. The *F*_*ST*_ values with and without application of the Excluding Null Alleles correction method were comparable (0.0068 vs. 0.0062, respectively, with overlapping 95% CI). After the removal of locus HGA8, no consistent support for linkage disequilibrium among the pair of loci and no significant deviation from Hardy-Weinberg equilibrium (Supplementary Table 2) were observed, as revealed by Fisher’s exact test.

Among the 14 loci examined, all were polymorphic with 6 to 12 alleles per locus, with an average of 9 alleles per total dataset (Supplementary Table 2). Expected (*H*e) and observed (*H*o) heterozygosity showed moderate variation among populations and ontogenetic stage (adults vs. juveniles), ranging from 0.63 to 0.77 (Table 2). Disregarding the population with the smallest sample size (IO_PA), the highest genetic diversity was observed in populations sampled in the mid Adriatic (AD_VI_A and AD_AN_J) and in the population sampled at the boundary of north-mid Adriatic (AD_LO_A). The effective number of alleles (Ae) and allelic richness (Ar) indices showed similar levels of diversity among populations and stages. Still, the number of alleles per locus was slightly reduced in juvenile stage of populations, likely affected by the limited sample sizes. Low and statistically insignificant values of the inbreeding coefficients, *F*_*IS*_, were recorded in the dataset (Table 2).

**TABLE 2:** Summary of genetic variation statistics for 14 microsatellites loci of European lobster *Homarus gammarus* in the central and eastern Mediterranean.

Pop ID	A	Ae	Ar	Ho	He	*F*_*IS*_
**Adults**						
AD_VE_A	6 ± 1.5	3.2 ± 1.1	3.5 ± 0.7	0.66 ± 0.1	0.67 ± 0.1	0.007
AD_UM_A	6.8 ± 1.2	3.5 ± 1.1	3.6 ± 0.7	0.67 ± 0.2	0.68 ± 0.1	0.018
AD_PU_A	7 ± 1.8	3.5 ± 1.3	3.7 ± 0.8	0.68 ± 0.1	0.68 ± 0.1	0.008
AD_LO_A	7.1 ± 1.6	3.7 ± 1.3	3.8 ± 0.8	0.70 ± 0.2	0.70 ± 0.1	0.001
AD_AN_A	5.5 ± 1.4	3.3 ± 1.1	3.6 ± 0.7	0.67 ± 0.2	0.69 ± 0.1	0.032
AD_HV_A	6.8 ± 1.8	3.6 ± 1.4	3.6 ± 0.8	0.67 ± 0.1	0.68 ± 0.2	0.006
AD_VI_A	6.6 ± 1.7	3.5 ± 1.3	3.7 ± 0.9	0.70 ± 0.2	0.68 ± 0.2	0.034
LG_CO_A	6.7 ± 1.7	3.4 ± 1.2	3.7 ± 0.7	0.68 ± 0.1	0.68 ± 0.1	0.002
IO_PA_A	3.9 ± 0.9	2.9 ± 0.9	3.7 ± 1.1	0.77 ± 0.2	0.73 ± 0.2	0.062
AE_AL_A	5.6 ± 1.8	3.1 ± 1.3	3.4 ± 0.9	0.64 ± 0.2	0.63 ± 0.2	0.018
*Overall*	8.9 ± 2.1	3.6 ± 1.2	3.6 ± 0.1	0.68 ± 0.1	0.68 ± 0.1	0.03
**Juveniles**						
AD_UM_J	4.4 ± 1.3	3.0 ± 1.1	3.4 ± 0.9	0.63 ± 0.2	0.66 ± 0.2	0.036
AD_PU_J	6.0 ± 1.4	3.3 ± 1.1	3.6 ± 0.7	0.63 ± 0.1	0.68 ± 0.1	0.077
AD_AN_J	5.9 ± 1.4	3.1 ± 0.8	3.6 ± 0.6	0.72 ± 0.1	0.68 ± 0.1	0.055
*Overall*	6.9 ± 1.5	3.3 ± 1.0	3.5 ± 0.1	0.66 ± 0.1	0.67 ± 0.1	0.027

*A, average number of alleles; Ae, effective number of alleles; Ar, allelic richness; He, expected heterozygosity; Ho, observed heterozygosity; F_IS_, fixation index. The last letter in the Pop ID abbreviation denotes the ontogenetic state of individuals (A, adult; J, juvenile).*

For several individual heterozygosity estimates, i.e., standardized observed heterozygosity per individual (Hs_exp, HS_obs), internal relatedness (IR) and homozygosity by locus (HL), no statistically significant differences (Mann–Whitney *U* test, *p* > 0.05) were detected between female and male individuals sampled in the Adriatic Sea (data not shown). The proportion of heterozygous loci was uncorrelated with carapace length (i.e., lobster age) for both female and male individuals (*p* > 0.05; Supplementary Figure 2).

### Between-Population Genetic Differentiation

**FIGURE 2:**
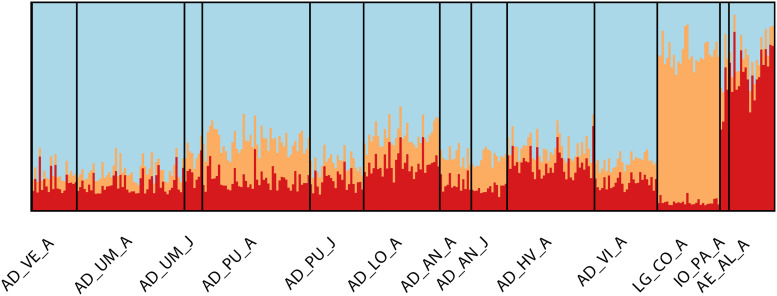
Bayesian clustering of European lobster *Homarus gammarus* populations in accordance to STRUCTURE assignment scores, where three (*K* = 3) inferred clusters were assumed. See Table 1 for additional information on sample names.

**FIGURE 3:**
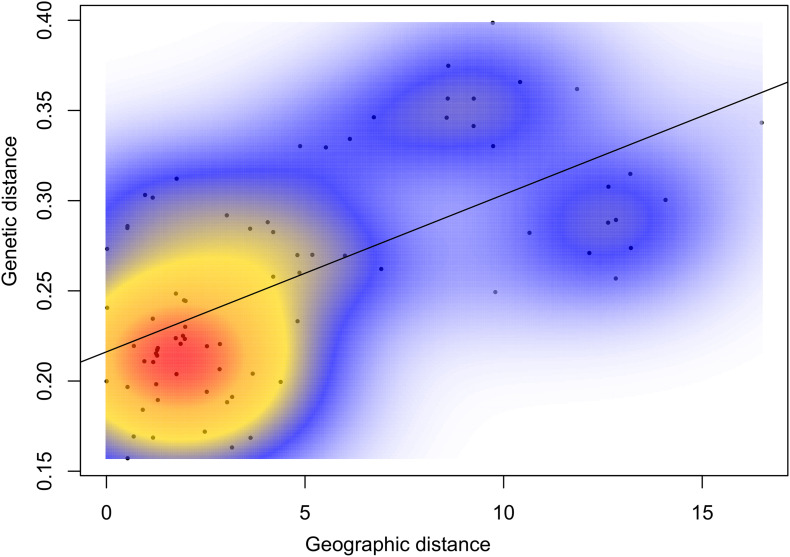
Isolation by distance plot showing the pairwise relationships of genetic distances in respect to the pairwise geographical distances in European lobster *Homarus gammarus* populations. Two-dimensional kernel density estimation in the MASS package in R was applied for the plot illustration, where the different colors present different correlation densities of genetic and geographic distances (red, high density; blue, low density).

Global genetic differentiation estimated with *F*_*ST*_ and *D*est was 0.0062 (*p* < 0.001) and 0.023, respectively, supporting a relatively limited level of differentiation across all populations. Following Bonferroni correction, both estimators gave congruent results in terms of pairwise comparisons, where 17 of 79 interactions were statistically significant (Table 3). On average, *D*est values were 1- to 2-fold higher than the equivalent statistic. High and significant pair-wise interactions were observed among populations from different Mediterranean basins. While the majority of Adriatic populations showed restricted gene flow toward the Aegean and Ligurian Sea populations, only the population from the western middle Adriatic and groups of juveniles from the northern Adriatic had no significant pair-wise differentiation from the population in the Ligurian Sea. Additionally, no breaks in gene flow were found within populations from the Adriatic, except for the populations sampled on the eastern side of the Adriatic, where the Vis Island population (AD_VI) sampled from the outermost point in the middle region showed significant interaction with the Lošinj Island population (AD_LO) from the north region. According to the mean L(K) and delta K statistics, three discrete genetic clusters of European lobster were inferred through Bayesian clustering analysis (Figure 2 and Supplementary Figure 3). All Adriatic populations were grouped into the first cluster with an average assignment score of 71%. The Ligurian Sea population formed a second cluster (82%) while the third cluster was associated with the Aegean Sea population (63%). The Ionian population showed an admixture proportion of membership in each of the three clusters. To explore potentially fine-scale population structure within the Adriatic Sea, a hierarchical STRUCTURE analysis was conducted exclusively on these populations. However, the mean L(K) suggested panmixia since the most likely number of populations was identified as *K* = 1 (data not shown). Presence of three clusters was also supported by the optimal number of clusters identified using DAPC and successive K-means clustering analyses. When each location was treated as an *a priori* cluster, the DAPC plot clustered groups similarly to the structure pattern noted in STRUCTURE (Supplementary Figure 4) where the gradual transition of clusters across the Adriatic, Ionian and Aegean Sea directions is seen in the PC space. Moderate isolation by distance was recorded by IBD analysis (*r* = 0.63, *p* = 0.01). Scatterplot of local densities of distances showed a patched form of genetic differentiation among populations due to the multiple densities of genetic relatedness with spatial discontinuities (Figure 3). Further analysis performed exclusively on the Adriatic Sea populations failed to detect IBD as a result of cline (*r* = 0.01, *p* = 0.78).

**TABLE 3:** Pairwise *F*_*ST*_ values (below diagonal) and Dest (above diagonal) based on 14 neutral microsatellites loci among 10 populations of European lobster *Homarus gammarus* including adult and juvenile individuals.

	AD_VE_A	AD_UM_A	AD_PU_A	AD_LO_A	AD_AN_A	AD_HV_A	AD_VI_A	LG_CO_A	IO_PA_A	AE_AL_A	AD_UM_J	AD_PU_J	AD_AN_J
AD_VE_A		0.012	0.000	0.013	0.013	0.001	0.005	0.045	0.013	0.055	0.005	0.012	0.002
AD_UM_A	0.006		0.010	0.013	0.009	0.003	0.009	0.042	0.042	0.061	0.009	0.004	0.001
AD_PU_A	0.000	0.005		0.010	0.016	0.007	0.010	0.021	0.032	0.052	0.004	0.001	0.005
AD_LO_A	0.006	0.006	0.005		0.007	0.005	0.024	0.057	0.045	0.038	0.002	0.010	0.010
AD_AN_A	0.005	0.003	0.007	0.003		0.014	0.009	0.015	0.037	0.052	0.046	0.029	0.027
AD_HV_A	0.000	0.001	0.003	0.002	0.006		0.004	0.036	0.043	0.041	0.018	0.008	0.002
AD_VI_A	0.003	0.004	0.004	0.010	0.004	0.002		0.045	0.017	0.047	0.002	0.006	0.014
LG_CO_A	0.022	0.020	0.010	0.025	0.008	0.017	0.021		0.024	0.107	0.010	0.021	0.007
IO_PA_A	0.008	0.021	0.017	0.023	0.018	0.023	0.012	0.013		0.017	0.056	0.033	0.043
AE_AL_A	0.029	0.030	0.026	0.018	0.027	0.021	0.023	0.054	0.011		0.026	0.061	0.063
AD_UM_J	0.004	0.004	0.002	0.001	0.021	0.009	0.001	0.006	0.030	0.015		0.027	0.017
AD_PU_J	0.005	0.002	0.000	0.005	0.012	0.003	0.004	0.010	0.015	0.032	0.011		0.018
AD_AN_J	0.002	0.001	0.003	0.004	0.013	0.000	0.005	0.002	0.026	0.031	0.009	0.008	

*Significant F_ST_ values are underlined at p < 0.001 (Bonferroni correction). Population codes are explained in Table 1.*

### Demographic Pattern and Gene Flow

No significant heterozygosity excess was observed under the two-phase model (TPM) parameters. In addition, the M ratio test showed no signal of a recent reduction in effective size, considering that the observed mean M-ratio of 0.77 ± 0.02 for the whole dataset was higher than the 0.68, i.e., the generally accepted threshold criterion in conservation genetics (Garza and Williamson, 2001; Hoban et al., 2013). While the majority of sampled populations had estimated M-ratio values (0.77–0.80) above their critical *M* values, two populations from the Adriatic Sea (AD_PU and AD_VI) showed evidence of a recent size reduction, but exclusively for ancestral theta (θ) values of 0.5 (Supplementary Figure 5).

**FIGURE 4:**
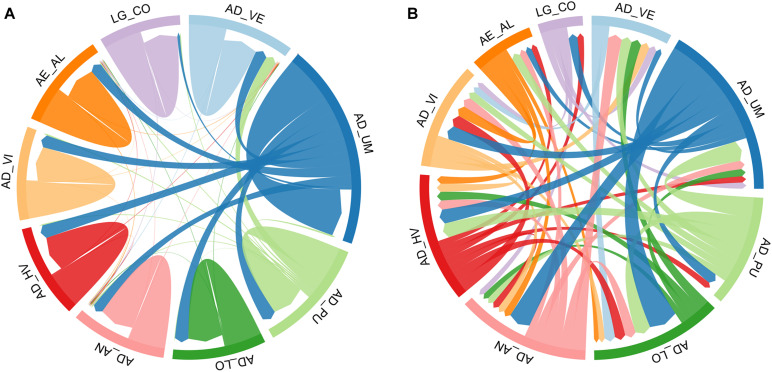
Gene flow diagrams for European lobster *Homarus gammarus* populations presenting **(A)** contemporary gene flow estimates (m) from BayesAss, and **(B)** historical gene flow estimates (M) from Migrate-n. Grid width represents the total amount of incoming and outgoing gene flow estimated for each population. Arrows indicate the direction of gene flow among the populations while the width of arrows are proportional to the relative amount of gene flow observed among connected populations. The wider arrow is linked with higher gene flow. The threshold for graphical illustration of gene flow was set to 0.01 for BayesAss and 2.0 for Migrate-n estimates. Exact gene flow estimates are presented in Supplementary Table 3. Populations codes are showed in Table 1.

Estimates of contemporary gene flow with BayesAss showed that populations in the north Adriatic (AD_UM and AD_LO) were the predominant source of exogenous allelic variants, with estimated emigration rates ranging from 0.06 for the most distant Corsica (LG_CO) population from the Ligurian Sea to 0.26 for the neighboring Lošinj (AD_LO) population from the north Adriatic (Figure 4A and Supplementary Table 3). In addition, there were no indications that the Umag population (AD_UM) in the north Adriatic received reproducing immigrants from other populations. Among other locations, little gene flow was detected, suggesting that these act as sink populations since the most of migration estimated values had 95% confidence intervals that overlapped zero.

Historical gene flow values using Migrate-n ranged from 0.83 to 11.3 with slightly more complex interactions between and within populations than contemporary gene flow estimates (Figure 4B and Supplementary Table 3). Again, the population from Umag (AD_UM) and less pronounced Pula (AD_PU) population acted as the main source of gene flow, supporting asymmetrical migration from the north toward the southern regions of the Adriatic. Still, migration rate between geographically distant clusters, as was the case for the Aegean Sea (AE_AL) and Ligurian Sea (LG_CO) populations tended to be lower, which is in line with local oceanographic features and relevant barriers. Gene flow among different basins was only evident in the case of the Aegean Sea population (source population) and populations from the eastern middle Adriatic (AD_VI and AD_HV; sink populations). Overall, historical migration rate estimates support more gene flow among all regions.

The contemporary effective population size, *N*e, of European lobster ranged from 74 (AD_HV) to 43,751 (AD_UM), but for all estimates the upper confidence limit reached infinity, indicating insufficient sample size to capture the genetic drift signal (Table 4). *Ne* values with both lower and upper 95% confidence bound were only captured for the Hvar population (AD_HV) in the mid Adriatic and for overall estimates, i.e., pooled Adriatic samples (2094) and the whole dataset (1560). The likelihood method implemented in AgeStructure (Wang et al., 2010) used to estimate the effective size for populations with overlapping generations, gave an *Ne* of 1651 (95% CI: 656–2051) for the pooled Adriatic populations; this value was slightly lower than the value estimated by the LD method where the 95% confidence intervals (CI) partially overlapped (Table 4). The paternal generation interval (GI) of 11.42 (95% CI: 9.81–12.67) and maternal GI of 12.74 (95% CI: 11.24–12.88) were estimated for the pooled Adriatic populations of known sex and size. Distribution of effective numbers of breeders of each age class varied between the sexes, where on average females had 20% higher values of breeders than males (Supplementary Table 4). Mutation scaled effective population size (Θ) calculated in Migrate-n ranged from 0.7 (AD_HV) to 1.8 (AD_UM), indicating that north Adriatic populations are effectively the largest. Still, these values should be scaled to the mutation rate for interpretation of *N*e estimates. To date, there are no published mutation rates for decapod microsatellites. Applying the mutation rate recorded in an orthopteran insect (phylum Arthropoda, Chapuis et al., 2015), the estimated theta values would correspond to *N*e values ranging from 791 (AD_HV) to 2087 (AD_UM) (Table 4).

**TABLE 4:** Contemporary, historical and age-structured effective population size (*N*e) with 95% confidence intervals (CI) for European lobster *Homarus gammarus* populations based on 14 putatively neutral microsatellite loci.

Pop ID	Contemporary *N*e (95% CI)	Theta (95% CI)	Historical *N*e
			
AD_VE	251 (61, ∞)	1.005 (0.449–1.529)	1196.4
AD_UM	43751 (333, ∞)	1.753 (1.129–2.349)	2087.6
AD_PU	3192 (407, ∞)	1.451 (0.889–1989)	1728.4
AD_LO	427 (137, ∞)	1.220 (0.489–1.729)	1453.0
AD_AN	∞ (212, ∞)	1.155 (0.589–1.689)	1376.1
AD_HV	74 (53,117)	0.665 (0.120–1.170)	791.3
AD_VI	248 (87, ∞)	0.981 (0.330–1.609)	1167.8
LG_CO	∞ (154, ∞)	0.966 (0.369–1.530)	1149.6
IO_PA	∞ (195, ∞)	–	–
AE_AL	441 (64, ∞)	0.674 (0.129–1.182)	802.3
Overall*	2094 (1024, 107189)		
Overall**	1560 (987, 3450)		
**AgeStructure *N*e (95% CI)**			
1651 (656–2051)			

Contemporary Ne was estimated in the program NeEstimator V2, theta (Θ) and historical Ne were estimated using Migrate-n where the equation Ne = theta/4μ was used to calculated historical Ne, assuming a mutation rate (μ) of 2.1 × 10^–4^ per generation. The program AgeStructure was applied to estimate AgeStructure Ne of individuals from the Adriatic Sea with known length size and sex. Details are provided in section “Materials and Methods.” *Overall Ne for Adriatic samples; **Overall Ne for whole dataset; Population from Ionian Sea (IO_PA) was excluded from Migrate-n analysis due to the small sample size.

### mtDNA Haplotype Diversity and Population Expansion

**FIGURE 5:**
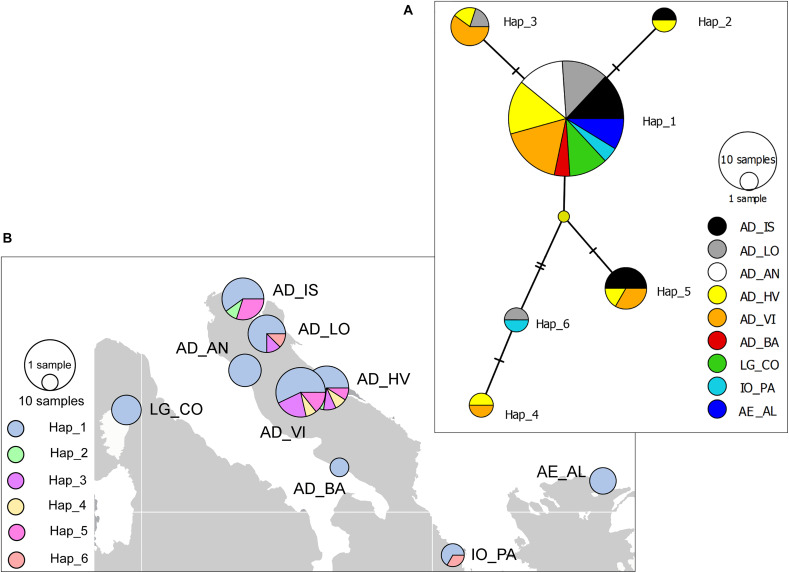
Median-joining network based on COI haplotypes **(A)** and geographical distribution of haplotypes **(B)** for European lobster *Homarus gammarus*. Colored circles represent haplotypes found in sampled populations whose sizes are proportional to the number of individuals. Missing haplotypes are indicated by small yellow dots. The pie charts on the map display the haplotype frequencies found at each location. Populations codes are given in Table 1.

Due to the poorly amplified products and discrepancies between forward and reverse sequences, the study design resulted in an unbalanced number of samples among locations. Thus, locations from the north Adriatic (AD_UM, AD_PU) were pooled into a single population (AD_IS), the Venice location was excluded, and the location near Bari (AD_BA) was introduced in dataset but with a limited number of individuals (Table 1). A 574-bp segment of the mitochondrial COI region was obtained, and 6 haplotypes were identified in 63 individuals (GenBank acc. no. MT983889 – MT983894). The number of haplotypes per population ranged from 1 to 5 (Figure 5). Haplotype diversity (Hd) averaged 0.46 across the five populations, and was higher for the middle Adriatic populations (0.626) than those in the north (0.44). The most common haplotype (Hap_1) was found at all sampling locations (Figure 5). The most distantly related rare haplotype (Hap_4) belonged to populations from the middle Adriatic (AD_HV and AD_VI) and was connected with the most common haplotype by a median vector representing the unsampled ancestral haplotype, most likely due to the limited sample size. Tajima’s D and Fu’s *Fs* test values for overall samples were negative but not significant, where mismatch distribution analysis and SSD test supported the hypothesis of a sudden population expansion (Supplementary Table 5). The non-significant values of neutrality tests probably reflect the limited power of these tests to detect departures from equilibrium, owning to the small sample size. Based on mutation rates ranging from 1.66 to 2.33% per million years, tau values calculated from the mismatch distribution of overall sequences from the Adriatic Sea provided estimates of coalescence in the range of 13.5–18.9 ka BP.

## Discussion

Knowledge of species distribution, population structure and size in relation to habitat suitability across local and broad spatial scales are key parameters for sustainable and ecosystem-based fisheries management. This is especially important for the semi-enclosed Mediterranean Sea, where intense fisheries effort has been recognized as one of the main stressors on the marine ecosystem (Coll et al., 2012; Colloca et al., 2013; Corrales et al., 2018). Namely, majority of marine stocks are considered fully exploited or overexploited (Vasilakopoulos et al., 2014; Osio et al., 2015; Tsikliras et al., 2015; FAO, 2018). Nowadays, European lobster stocks are considered depleted due to significant declines in catches in many Mediterranean regions (Lloret and Riera, 2008; Lotze et al., 2011; Pere et al., 2019), although stock assessment has not been performed yet. To support genetically informed European lobster fisheries management in the Adriatic Sea, 14 neutral microsatellites loci and partial mtDNA region were used to explore fine-scale genetic structure of species and migration patterns that affect genetic connectivity, by comparing 331 individuals sampled from the central and eastern regions of the Mediterranean. To the best of our knowledge, this is the first description of the genetic diversity and population structure of the European lobster *H. gammarus*, a decapod crustacean species of ecological and economic importance, in the waters of the Adriatic Sea.

Previous studies have explored European lobster population structure at different geographical scales using novel and traditional markers, i.e., RAPDS (Ulrich et al., 2001), allozymes (Jørstad et al., 2005), mtDNA and RFLPs (Triantafyllidis et al., 2005), microsatellites (Huserbråten et al., 2013; Watson et al., 2016; Ellis et al., 2017) and SNPs (Jenkins et al., 2019, 2020). At the broad geographical scale, coherent patterns of gene flow discontinuity were observed where several major distinct groups, i.e., Atlantic, northern Norway, Netherlands and Mediterranean were identified with the presence of an IBD pattern at the European level (for more detail see Triantafyllidis et al., 2005; Ellis et al., 2017). Both authors suggest that populations located toward the distribution limits, as in the case for populations originating from northern Norway and the Aegean Sea, show discrete biological units due to reduced migration rate and consequently increased genetic drift. Additionally, mtDNA analysis also supported sub-regional division within the Mediterranean, confirming differentiation of the Aegean populations from Adriatic and western Mediterranean ones (Triantafyllidis et al., 2005). This pattern was also observed in the present work, where the highest level of genetic differentiation was found among populations from different Mediterranean basins, while populations from the Adriatic Sea proved to be panmictic. Aside from the low level of overall genetic differentiation of European lobster in the present dataset (*F*_*ST*_ = 0.0062) that overlapped with the level previously documented using the same set of microsatellite loci (*F*_*ST*_ = 0.007; Ellis et al., 2017) on a wide sample collection throughout Europe, the majority of *F*_*ST*_ pair-wise comparisons among Adriatic-Aegean and Adriatic-Ligurian populations were significantly different. High gene flow and low values of *F*_*ST*_ in marine species do not mean an absence of population structure, because exchange of only few individuals per generation among populations can maintain genetic homogeneity (Waples, 1998). High gene flow documented in the Adriatic Sea and reduced flow toward other basins has been verified by both spatial analyses suggesting larval retention and confined dispersal within the Adriatic Sea. Namely, hierarchical Structure and DAPC analysis gave three distinct clusters assigning all the Adriatic populations into the first cluster, populations from the Ligurian and Aegean Seas into the second and third clusters, with the exception of the Ionian population that showed a more heterogeneous origin. It seems that the semi-enclosed Adriatic Sea presents one of defined phylogeographic regions within the Mediterranean. Namely, reduced gene flow has been documented for several other marine species (*Ruditapes decussatus*, Cordero et al., 2014; *Sepia officinalis*, Pérez-Losada et al., 2007; *Solea solea*, Sabatini et al., 2018; *Paracentrotus lividus*, Maltagliati et al., 2010) between the Adriatic Sea and the rest of Mediterranean.

Still, a diffuse barrier between the Adriatic and Ionian coast was observed in the present study although due to the limited sample size of the Ionian group, no strong conclusions can be drawn regarding population connectivity. The Ionian population showed a north-to-south transition pattern of differentiation characterized with low and non-significant *F*_*ST*_ pair-wise comparisons with all other sampled groups. Furthermore, all individuals in that group were partially assigned to all three clusters identified by Bayesian analysis. The Ionian Sea presents no phylogeographic barriers and is linked with the Adriatic Sea via oceanographic processes (Villamor et al., 2014), primarily coastal marine currents. Therefore, larval exchange and contemporary gene flow greatly depends on the dispersal capacity of the species (Rossi et al., 2014).

The most divergent individuals characterized by high membership coefficients of its assigning cluster originated from the Ligurian Sea. This differentiation seems to be result of significant IBD. Still, instead of a continuous cline of genetic differentiation, our data revealed a discontinuity in the form of patches, indicating the presence of barriers to gene flow or pattern potentially biased with not unformal sampling distribution across the study region (Meirmans, 2012). Nevertheless, we argue that in the case of the Ligurian population, IBD is a less important driver of the contemporary population structure. Limited gene flow toward the central Mediterranean can be partially explained by the well-defined Siculo-Tunisian Strait, an ocean front identified as a barrier for gene flow in some marine species (i.e., Mejri et al., 2009). In addition, recent study based on 79 SNPs analysis suggested that the hatchery stocking program conducted in Corsica during the 1970s, using lobsters originated from the Atlantic coast of France, left the footprint of Atlantic ancestry in the contemporary population and impacted the genetic structure of the Corsican natural population (Jenkins et al., 2020). Such a restocking impact by European lobster of Atlantic origin may additionally explain the observed discontinuity of genetic differentiation within the Mediterranean. Similar intraspecific hybridization pattern between escaped farmed fish of Atlantic origin and wild conspecifics has already been documented in the Adriatic where highly admixed populations had decreased genetic diversity (Šegvić-Bubić et al., 2017; Žužul et al., 2019).

The study results suggest that observed reductions in population size due to overfishing, habitat loss and other anthropogenic activities have not significantly impacted the neutral genetic diversity. The results of the heterozygote excess test under the TPM model showed no genetic erosion signal in all tested populations of *H. gammarus*, while M ratios were above 0.68 (average 0.77) as the cut off value for selecting demographically stable natural populations (Garza and Williamson, 2001). Genetic diversity indices for both juveniles and adults were comparable to values reported for European lobster populations from northern European waters (Ellis et al., 2017) where the same microsatellites markers were employed. Additionally, the results corroborate with those observed in a population from Lundy Island, a marine protected area in the Bristol Channel (United Kingdom) established in 2003 (Watson et al., 2016), suggesting stability of genetic variability despite intense fishing effort within the regions. Still, there is the potential limitation of the present data set to detect bottleneck events due to the relatively short time since the significant decrease in the census size of European lobster in the Adriatic Sea. Namely, the last significant decrease in landings was recorded in the 1990s, from 119 t y^–1^ to 10 t y^–1^ afterward (Lotze et al., 2011). Taking into account species longevity (42–72 years, Sheehy et al., 1999) and the relatively long generation time (9.81–12.88 years, present study), it could be argued that too few generations have passed to observe any loss of generation diversity by drift.

Contemporary and historical gene flow estimations demonstrated limited exchange of genetic material between basins, but not within the Adriatic Sea. Namely, both Migrate and BayesAss results showed that the north eastern coast of the Adriatic Sea acts as the main source of gene flow in the Adriatic Sea, with an asymmetrical migration pattern from the north toward the southern regions, which was additionally supported by the lack of IBD within the basin. Even though the model diagnostics for these analyses and model convergence performed well, the BayesAss method has reduced sensitivity when *F*_*ST*_ values between populations are less than 0.05, or in cases of high migration rates (Faubet et al., 2007). Since global and pairwise *F*_*ST*_ values among sampled populations were below the method-supported threshold, caution is required in interpreting these results as precise migration values. Galparsoro et al. (2009) produced the modeled habitat suitability map for the Bay of Biscay and pointed out that the most favorable habitat for European lobster are locations at the boundary between sedimentary and rocky-bottoms at water depths of 35–40 m. The eastern coast of the northern Adriatic meets these conditions. The Adriatic Sea is classified into three sub-regions, where depth increases from north to south (Trincardi et al., 1994). The northern region is the most extensive continental shelf of the Mediterranean Sea with an average bottom depth of about 35 m (Trincardi et al., 1994). The eastern coast of the north region, where the source populations (AD_UM, AD_PU) were sampled, is steep, with minimal recent sedimentation and strong karst relief that influences sediment distribution patterns (Fütterer and Paul, 1976). In contrast to the middle and south regions, which are characterized by average depths of 150 m and broad depressions 1218–1225 m deep, the heterogeneous bottom and relatively shallow depths makes the eastern coast of the north region the most suitable habitat, as the rocky substrate provides shelter from predators while soft sediment increases food availability for the European lobster. Since adult lobsters show high site fidelity in multiple locations (Øresland and Ulmestrand, 2013; Wiig et al., 2013; Skerritt et al., 2015), it would appear that gene flow within the Adriatic relies on dispersal potential of planktonic larvae with the support of local oceanographic features.

The circulation in the Adriatic is mostly counter-clockwise or cyclonic, with currents that northerly inflows along the eastern coast and returns in a southerly direction along the western coast, with up to pronounced circulation cells in the southern, middle and northern region (Orlić et al., 2012). Over a general pattern of panmixia, such circulation system greatly affects seascape connectivity in the Adriatic (Melià et al., 2016) and may be responsible for slight gene break observed between Vis Island population (AD_VI) from the middle region and the Lošinj Island population (AD_LO) from the north region. Although self-recruitment at a small spatial scale has been reported elsewhere (Øresland and Ulmestrand, 2013; Watson et al., 2016), we postulate that the Western Adriatic Current supports larval dispersal from the northern Adriatic, enabling genetically homogenized populations in the core of their range. Taking the duration of pelagic larval duration into account, i.e., 2 to 4 weeks (Schmalenbach and Franke, 2010) and an average current velocity of 25 cm/s (Poulain, 2001), the transfer of genetic material could potentially occur at a relatively large spatial scale, up to distances of 300 to 600 km. Due to the temporal fluctuation of population connectivity, replicated sampling is needed, including populations at the southern-most limits of basin to be able to estimate genetic identity of all possible source populations within the Adriatic Sea.

The mean contemporary *N*e estimated for European lobster populations varied from 1,560 to 2,094, depending on the method employed, with overlapping CI of these values with historical estimations per population. To cope with the bias introduced by overlapping generations on estimates by the LD method (NeEstimator; Do et al., 2014), another option, i.e., estimator by parentage assignments (AgeStructure; Wang et al., 2010), specifically developed for populations with overlapping generations was used to estimate *N*e by engaging only individuals of known age and sex. These two different approaches gave congruent estimations of *N*e for the Adriatic basin, thereby increasing confidence in the estimates. Interestingly, the AgeStructure analysis revealed a decreased *N*e per age class for males up to 20% in contrast to the *N*e of females. Due to behavioral differences between the sexes, males are less residential and use more space than females (Skerritt et al., 2015), and as such are more vulnerable to fishing than females. This can affect census size and consequently *N*e of males. A similar overall *N*e estimate was observed in European lobsters from northern European waters (Ellis et al., 2017). Additionally, these *N*e values fall within the range of the critical population size (*Ne*≃1000–5000) necessary for the maintenance of sufficient genetic variance for adaptive response in quantitative traits (Bürger and Lynch, 1997). It seems that the European lobster maintains adequate power to respond to environmental challenges, as already seen in some other overfished marine species (Riccioni et al., 2010; Valenzuela-Quiñonez et al., 2014).

Average haplotype diversity (0.456) across investigated populations was consistent with data for other marine crustaceans in the Mediterranean (Deli et al., 2015; Marra et al., 2015) but lower than those reported from European lobster populations examined using RFLP analysis of a 3-kb mtDNA fragment (Triantafyllidis et al., 2005). The same authors observed reduced genetic diversities in populations from the Adriatic and Aegean Seas in comparison with other Mediterranean regions, stressing that a possible cause is the synergistic effect of genetic bottleneck, absence of immigration that would increase genetic variation and high exploitation. Still, diversity indices from the present data are not comparable with these studies, given the use of different markers and/or sequence length and the lack of data robustness. Nevertheless, the hypothesis of sudden population expansion was not rejected for the present COI dataset and the expansion time estimates supports the hypothesis of a Pleistocene event, i.e., the Last Glacial Maximum (LGM ∼ 18 ka BP) for the Adriatic Sea, where expansion and contraction of European lobster populations were impacted by sea-level (Svitoch et al., 2000). The northern and central regions of the Adriatic were then dry and the sea level was around 130 m lower than today (Malvić and Velić, 2011; Sikora et al., 2014). Subsequently, the south-north orientation of the basin contributed to the postglacial recolonization of the Adriatic from southern refugia in the northward direction (Bianchi et al., 2012). Serial founder events along the recolonization axis may have led to a reduced genetic diversity at the edges of expansion range due to genetic drift. The impact of such a recolonization dynamic on genetic diversity was suggested for the violescent sea-whip *Paramuricea clavata* in the Eastern Adriatic (Ledoux et al., 2018). In this study, the different haplotype diversity levels observed between the north (0.44) and middle Adriatic (0.62) could be explained by the same factors.

### Management Implications

Knowledge about stock structure and population connectivity is important for effective fisheries management. For the first time, this study provides detailed genetic structure of the European lobster in the Adriatic Sea and information on population connectivity between the Adriatic and Mediterranean basins. The European lobster shows signs of overexploitation in the Croatian fishery (Lotze et al., 2011). Despite the implementation of minimum legal size-limit and restricted fishing periods as regulatory measures for lobster fisheries, no additional strategic or long-term management actions have been proposed to improve the species abundance. The improvement of productivity and sustainability of European lobster fisheries in Europe has been practiced through two approaches: lobster stocking (Ellis et al., 2015b) and establishment of marine protected areas (MPAs) (Nillos Kleiven et al., 2019). While impact assessment and potential for lobster stocking remains limited, partially due to unfavorable juvenile tagging methods and limited information on species ecology, MPAs have demonstrated potential in lobster sustainable management. Namely, after 4 years of MPA establishment in southern Norway, CPUE values from experimental fishing had increased by a magnitude of 2.6 compared to pre-protection values (Nillos Kleiven et al., 2019). Thus, we propose establishment of new interconnected MPAs, targeting primarily the eastern part of north Adriatic, an area recognized as the main source of gene flow in the present study. Aside from the already active MPAs, such as the Brijuni archipelago in the north region, a large proportion of lobster habitat remains vulnerable to anthropogenic disturbance and the current level of protection of 3% for the north Adriatic Sea seems insufficient (Bastari et al., 2016). To achieve successful biodiversity protection, marine reserves should be primarily connected to other MPAs via adults or larval dispersal. Thus, species biological traits knowledge such as the pelagic duration and the spawning pattern, along with Lagrangian particle transport modeling within the local current system to study larval transport and population connectivity should be considered for adequate spatial conservation planning (Bray et al., 2017; Legrand et al., 2019).

While sign of neutral genetic diversity loss was not observed for European lobster populations in the present study, fishery and other impacts may have caused changes in the frequency of selectively important traits that cannot be easily measured with present genetic markers. Additionally, an important limitation of assignment methods used here is their power to detect migrants in cases of low levels of population differentiation, although it can be counterbalanced by larger sample sizes and additional gene loci, resulting in suitable power to detect migrants (Paetkau et al., 2004; Manel et al., 2005). In most cases, marine species function as discrete populations in the adult phase, connected with a dispersive pelagic larval stage and sea currents where high gene flow and associated lack of population structure can be expected when using neutral microsatellite markers. The priority for fisheries and conservation management to recognize source-sink attributes of fish populations, has invoke increase of population genomics studies and use of single-nucleotide polymorphism (SNP) markers. Many marine species showed substantial levels of differentiation at putative candidate loci under selection (Cure et al., 2017), providing new insights into source population and recruitment event connection.

Recently developed SNP markers using RAD sequencing have proven to be powerful in population genetic structure detection (Jenkins et al., 2019). Still, the findings of this study provide novel insights into the genetic diversity and structure of the European lobster in the Adriatic Sea and will serve for fisheries management and the spatial planning of marine reserve networks. Additional efforts will be oriented toward use of SNP markers and researching the genetic architecture of quantitative traits to examine the consequences of fisheries-induced selection on recent European lobster populations.

## Data Availability Statement

The datasets presented in this study can be found in online repositories. The names of the repository/repositories and accession number(s) can be found below: NCBI (GenBank acc. no. MT983889 – MT983894).

## Ethics Statement

Ethical review and approval was not required for the animal study because lobster tissue samples were obtained exclusively from commercial fisheries.

## Author Contributions

TŠ-B, SM-S, and MP conceived the study. MP, AT, FG, ED, and IC conducted the sampling. MP, IŽ, and TŠ-B conducted the molecular and data analysis. TŠ-B and MP wrote and revised the manuscript. SM-S obtained funding for the work. All authors have reviewed and approved the final manuscript.

## Conflict of Interest

The authors declare that the research was conducted in the absence of any commercial or financial relationships that could be construed as a potential conflict of interest.

## Footnotes

1 http://popart.otago.ac.nz
