# Supplementary material for: Population Genetic Structure and Connectivity of the European Lobster Homarus gammarus in the Adriatic and Mediterranean Seas
Source: Front Genet. 2020 Dec 7;11:576023. doi: 10.3389/fgene.2020.576023 (PMC7750201; doi:10.3389/fgene.2020.576023)
Supplement: Supplementary file 1 [file Data_Sheet_1.PDF]

Supplementary material

Heterozygosity measures estimation:

A total of 4 heterozygosity measures, i.e. standardized observed heterozygosity per individual (HS_obs), the internal relatedness (IR) and the homozygosity by locus (HL), for each individual with known length size and sex were estimated as the proportions of heterozygous genotypes using the function GENHET.R (Coulon, 2010) in the R (R Core Team, 2017).

a) *Standardized heterozygosity* based on the mean observed heterozygosity (Hs_obs) was calculated by dividing the proportion of heterozygous loci (PHt) in an individual with mean observed heterozygosity of typed loci. This estimate is highly conservative and perform well in cases of data exhibiting null alleles or allele drop-out (Amos 2005).
b) Standardized heterozygosity based on the mean expected heterozygosity (Hs_exp) was calculated by dividing the proportion of heterozygous loci (PHt) in an individual with mean expected heterozygosity of typed loci (Coltman et al. 1999).
c) *Internal relatedness* IR is a measure of parental relatedness that weighs heterozygosity by allele frequency. It is calculated with the formula: $2H-\sum fi/2N-\sum fi$, where H is the number of homozygous loci in an individual, N is the total number of loci, and f_i is the frequency of the ith allele in the genotype. IR assumes values between −1 (all heterozygous) and 1 (all homozygous). Negative IR values indicate more outbred and positive IR values suggest more inbred individuals within the studied sample (Amos et al. 2001).
d) Homozygosity by locus HL is calculated with the formula: $\sum Eh / \sum Eh+\sum Ej$, where Eh and Ej are the expected heterozygosities of the loci that an individual bears in homozygosis (h) and in heterozygosis (j). To take into account the fact that some loci are more informative than others, HL considers the contribution of each locus instead of the contribution of each allele (Aparicio et al., 2006).

Linear regressions was used to investigate relationships between measures of diversity and carapace length for both sexes.

Aparicio, J. M., Ortego, J., and Cordero, P. J. (2006). What should we weigh to estimate heterozygosity, alleles or loci? *Mol. Ecol.* 15, 4659–4665. doi:10.1111/j.1365-294X.2006.03111.x.

Coulon, A. (2010). genhet: an easy-to-use R function to estimate individual heterozygosity. *Mol. Ecol. Resour.* 10, 167–169. doi:10.1111/j.1755-0998.2009.02731.x.

R Core Team (2017). R: A language and environment for statistical computing. R Foundation for Statistical Computing, Vienna, Austria. Available at: https://www.r-project.org/ [Accessed July 1, 2019].
