# Supplementary material for: Population Genetic Structure and Connectivity of the European Lobster Homarus gammarus in the Adriatic and Mediterranean Seas
Source: Front Genet. 2020 Dec 7;11:576023. doi: 10.3389/fgene.2020.576023 (PMC7750201; doi:10.3389/fgene.2020.576023)
Supplement: Supplementary file 2 [file Data_Sheet_2.PDF]

Supplementary Table S1. Primer sequences, fluorescent dyes, linkage group and combinations of primers in three (M1, M2, M3) multiplex PCRs, number of alleles, type of nucleotide motif and the references for each microsatellite loci are provided. The locus HGA8 was omitted from further analyses. For more details, see the Results section.

Multiplex PCR	Locus/ Primer code	5'-3' sequence of the primer	5' fluorescent dye	Linkage group	Nucleotide motif	Allele size range (bp)	Number of alleles	Reference
M1	HGA8	F: TTGAACAGCAAAAACGTAGTG<br>R: ACATCACACCACAACTCACTG	6-FAM		Tetra	269–325	12	André and Knutsen (2010)
	HGB4	F: TTCGCTAGTCCGTCTGTCC<br>R: ACGAAGGATTACGGCACAT	NED		Tetra	187–231	6	André and Knutsen (2010)
	HGC118	F: TCGTTTCCAATGGTCTCG<br>R: AAGTTGAAGGAGGTGCTTGAC	VIC		Tetra	262-296	7	André and Knutsen (2010)
	HGD106	F: CATACCGAACCAAGTGTAAAC<br>R: GCCCACAGTAACAGATAAGAG	6-FAM		Tetra	139-167	7	André and Knutsen (2010)
	HGD117	F: GCCTACTCTCTCCTTCCTTC<br>R: CCTGTCTATCGTTCTGTTTG	PET		Tetra	254-302	10	Ellis et al. (2015)
M2	HGC103	F: TGGTATTATGGCTACGACAAG<br>R: CAAAAGACGGGTTTCAATC	6-FAM		Tetra	220-254	11	André and Knutsen (2010)
	HGB6	F: AGAAGGGAGGTGGGTGAG<br>R: ATGAACCCGTCTGAGGTTATC	VIC		Tetra	150-190	7	André and Knutsen (2010)
	HGC120	F: CCCTCTCTCATCCCTCTTATC<br>R: ACCCTTATTCATCCATCCTTC	VIC		Tetra	251-297	13	André and Knutsen (2010)
	HGD129	F: TTGAACGCTATGAACTGAGAC<br>R: AGGCATACAAATAAACGCAC	NED		Tetra	234-290	10	Ellis et al. (2015)
	HGC6	F: AGGCTGCATAGTTACACGTTTG<br>R: ACCCAGTGTCAAGGAATAGTCC	PET		Tetra	274-318	6	André and Knutsen (2010)
M3	HGD110	F: ACGGATGGATGGATAGGTAG<br>R: ATTCTCTGGCAGGTCAAGAC	6-FAM		Tetra	176-220	11	Ellis et al. (2015)
	HGC129	F: TTGAACGCTATGAACTGAGAC<br>R: AGGCATACAAATAAACGCAC	6-FAM		Tetra	247-291	6	André and Knutsen (2010)
	HGC131b	F: CATGGGTGATTAGGATGACC<br>R: TGGCACCATAGGTTCGTATC	VIC		Tetra	226-276	12	André and Knutsen (2010)
	HGC111	F: TGAAGCGTGGAGGACCTT<br>R: CACACCTGTCTGGCTACACC	NED		Tetra	160-190	10	André and Knutsen (2010)
	HGD111	F: TAAAGGTGATGTTCAGTCCAC<br>R: CTTGACCCGCTACCAATAC	PET		Tetra	231-275	8	André and Knutsen (2010)

Supplementary Table S2. Summery statistics of 14 neutral microsatellite loci among samples of European lobster *Homarus gammarus*.

Lo		AD_VE_A	AD_UM_A	AD_PU_A	AD_LO_A	AD_AN_A	AD_HV_A	AD_VI_A	LG_CO_A	IO_PA_A	EG_AL_A	AD_UM_J	AD_PU_J	AD_AN_J
**D106**	**n**	20	48	48	34	14	39	28	28	4	20	8	24	16
	**nA**	6	6	7	6	6	6	6	7	4	5	5	5	5
	**HO**	0.85	0.85	0.77	0.82	0.79	0.67	0.75	0.86	1.00	0.80	0.63	0.67	0.81
	**HE**	0.78	0.80	0.79	0.78	0.80	0.75	0.73	0.76	0.82	0.74	0.78	0.69	0.75
	**FIS**	-0.1	-0.07	0.02	-0.06	0.02	0.11	-0.04	-0.13	-0.26	-0.08	0.21	0.03	-0.08
**C118**	**n**	20	48	48	34	14	39	28	28	4	20	8	24	16
	**nA**	6	6	6	6	6	6	6	6	4	5	4	6	6
	**HO**	0.65	0.60	0.60	0.74	0.71	0.62	0.57	0.61	0.50	0.55	0.63	0.71	0.50
	**HE**	0.59	0.58	0.60	0.70	0.68	0.59	0.54	0.58	0.79	0.73	0.65	0.63	0.62
	**FIS**	-0.11	-0.05	0.06	-0.05	-0.05	-0.05	-0.06	-0.04	0.4	0.25	0.04	-0.13	0.2
**B4**	**n**	20	48	48	34	14	39	28	28	4	20	8	24	16
	**nA**	4	5	6	6	4	5	4	6	2	3	3	4	3
	**HO**	0.45	0.48	0.50	0.56	0.50	0.49	0.46	0.61	0.25	0.45	0.13	0.58	0.50
	**HE**	0.54	0.50	0.55	0.82	0.58	0.40	0.40	0.62	0.25	0.38	0.24	0.48	0.45
	**FIS**	0.17	0.03	0.09	-0.17	0.14	-0.21	-0.15	0.02	0	-0.19	0.5	-0.22	-0.13
**D117**	**n**	20	48	48	34	14	39	28	28	4	20	8	24	16
	**nA**	3	7	5	6	4	6	5	3	3	6	3	5	4
	**HO**	0.60	0.48	0.58	0.68	0.79	0.62	0.75	0.46	0.75	0.55	0.75	0.29	0.69
	**HE**	0.55	0.59	0.59	0.60	0.60	0.61	0.62	0.44	0.61	0.56	0.54	0.58	0.56
	**FIS**	-0.1	0.18	0.02	-0.14	-0.32	-0.01	-0.22	-0.05	-0.29	0.02	-0.42	0.50	-0.23
**C103**	**n**	20	48	48	34	14	39	28	28	4	20	8	24	16
	**nA**	6	7	6	6	5	5	6	5	3	4	4	6	5
	**HO**	0.70	0.71	0.73	0.65	0.71	0.82	0.79	0.64	0.75	0.60	0.75	0.71	0.75
	**HE**	0.64	0.69	0.70	0.72	0.70	0.73	0.69	0.70	0.71	0.63	0.64	0.71	0.72
	**FIS**	-0.09	-0.02	-0.04	-0.10	-0.01	-0.13	-0.14	0.08	-0.06	0.05	-0.18	0.01	-0.05
**C120**	**n**	20	48	48	34	14	39	28	28	4	20	8	24	16
	**nA**	7	9	10	9	6	9	8	7	5	6	4	7	6
	**HO**	0.70	0.75	0.81	0.74	0.71	0.69	0.71	0.79	1.00	0.55	0.63	0.54	0.75
	**HE**	0.79	0.80	0.84	0.78	0.77	0.81	0.78	0.86	0.86	0.59	0.78	0.82	0.79
	**FIS**	0.12	0.06	0.04	0.06	0.08	0.15	0.08	0.09	-0.2	0.07	0.21	0.34	0.05
**B6**	**n**	20	48	48	34	14	39	28	28	4	20	8	24	16
	**nA**	5	6	6	7	5	6	6	6	5	6	5	5	7
	**HO**	0.90	0.79	0.73	0.82	0.71	0.82	0.79	0.79	0.75	0.75	1.00	0.67	0.75
	**HE**	0.76	0.73	0.76	0.83	0.74	0.75	0.77	0.76	0.86	0.72	0.78	0.74	0.69
	**FIS**	-0.18	-0.08	0.04	0.01	0.04	-0.1	-0.02	-0.04	0.14	-0.04	-0.32	0.11	-0.08
**D129**	**n**	20	48	48	34	14	39	28	28	4	20	8	24	16
	**nA**	6	7	6	7	4	6	8	6	4	5	3	7	6
	**HO**	0.55	0.79	0.54	0.65	0.57	0.72	0.71	0.61	0.75	0.70	0.63	0.67	0.81
	**HE**	0.53	0.77	0.56	0.69	0.66	0.69	0.74	0.63	0.75	0.59	0.69	0.63	0.69
	**FIS**	-0.04	-0.03	0.03	0.07	0.14	-0.04	0.03	0.03	0	-0.19	0.10	-0.05	-0.19
**C6**	**n**	20	48	48	34	14	39	28	27	/	20	8	24	16
	**nA**	6	6	6	6	4	6	5	7	/	2	3	6	6
	**HO**	0.40	0.33	0.40	0.29	0.21	0.31	0.25	0.41	/	0.10	0.25	0.50	0.63
	**HE**	0.36	0.37	0.40	0.36	0.32	0.30	0.26	0.49	/	0.10	0.24	0.50	0.56
	**FIS**	-0.11	0.09	0.01	0.2	0.35	-0.01	0.06	0.18	/	-0.03	-0.04	0.01	-0.12
**C129**	**n**	20	48	48	34	14	39	28	28	4	20	8	24	16
	**nA**	8	6	9	8	7	10	9	7	5	7	5	8	6
	**HO**	0.70	0.67	0.75	0.74	0.57	0.72	0.71	0.71	0.75	0.75	0.63	0.75	0.75
	**HE**	0.81	0.73	0.77	0.82	0.77	0.83	0.79	0.71	0.79	0.77	0.79	0.83	0.73
	**FIS**	0.14	0.08	0.03	0.1	0.26	0.14	0.1	-0.001	0.05	0.03	0.22	0.1	-0.03
**D110**	**n**	20	48	48	34	14	39	28	28	4	20	8	24	16
	**nA**	6	8	9	10	7	8	8	9	4	8	7	5	7
	**HO**	0.85	0.73	0.77	0.88	0.71	0.74	0.86	0.71	1.00	0.80	0.88	0.54	0.81

	**HE**	0.71	0.76	0.78	0.78	0.77	0.77	0.77	0.78	0.75	0.69	0.79	0.71	0.74
	**FIS**	-0.2	0.05	0.01	-0.14	0.08	0.04	-0.12	0.09	-0.41	-0.17	-0.11	0.24	-0.1
**C131b**	**n**	20	48	48	34	14	39	28	28	4	20	8	24	16
	**nA**	9	9	10	10	8	10	9	10	4	9	7	8	9
	**HO**	0.65	0.83	0.77	0.79	1.00	0.79	0.96	0.79	0.75	0.80	1.00	0.75	0.88
	**HE**	0.81	0.82	0.80	0.85	0.86	0.86	0.86	0.79	0.75	0.87	0.87	0.80	0.70
	**FIS**	0.2	-0.02	0.04	0.07	-0.17	0.07	-0.12	0.01	0	0.08	-0.17	0.06	-0.1
**C111**	**n**	20	48	48	34	14	39	28	28	4	20	8	24	16
	**nA**	5	6	5	5	4	5	4	7	4	6	4	4	6
	**HO**	0.60	0.63	0.85	0.88	0.86	0.74	0.71	0.75	1.00	0.80	0.50	0.75	0.75
	**HE**	0.71	0.73	0.75	0.74	0.68	0.69	0.76	0.70	0.79	0.77	0.75	0.68	0.74
	**FIS**	0.16	0. 15	-0.14	-0.19	-0.27	-0.08	0.06	-0.08	-0.33	-0.04	0.35	-0.11	-0.01
**D111**	**n**	20	48	48	34	14	39	28	27	4	20	8	24	16
	**nA**	7	7	7	8	7	7	8	8	4	6	4	8	6
	**HO**	0.65	0.73	0.69	0.59	0.50	0.69	0.75	0.78	0.75	0.75	0.50	0.67	0.69
	**HE**	0.73	0.68	0.68	0.71	0.71	0.72	0.76	0.70	0.79	0.65	0.65	0.71	0.72
	**FIS**	0.11	-0.07	-0.004	0.18	0.3	0.04	0.01	-0.12	0.05	-0.17	0.24	0.06	0.05

Supplementary Table S3. (a) Contemporary gene flow with 95% confidence intervals for European lobster *Homarus gammarus* using BayesAss. The population from the Ionian Sea was excluded from the analysis due to small sample size. Source populations = rows, sink populations = columns. Population codes and colours used as in Figure 4.

a)

Pop ID	AD_VE	AD_UM	AD_PU	AD_LO	AD_AN	AD_HV	AD_VI	LG_CO	EG_AL
AD_VE	0.678 (0.66-0.70)	0.006 (0.0-0.02)	0.0058 (0.00-0.02)	0.0076 (0.0-0.02)	0.0144 (0.0-0.04)	0.007 (0.0-0.02)	0.0089 (0.0-0.03)	0.0091 (0.0-0.03)	0.0116 (0.0-0.03)
AD_UM	0.2329 (0.17-0.29)	0.9231 (0.87-0.98)	0.2792 (0.24-0.31)	0.2676 (0.23-0.31)	0.2063 (0.14-0.28)	0.2621 (0.22-0.31)	0.2432 (0.18-0.30)	0.0519 (0.0-0.10)	0.2293 (0.17-0.29)
AD_PU	0.2186 (0.15-0.28)	0.0353 (0.01-0.08)	0.6791 (0.66-0.70)	0.0118 (0.0-0.03)	0.026 (0.0-0.07)	0.0224 (0.0-0.06)	0.0282 (0.0-0.07)	0.0201 (0.0-0.05)	0.0237 (0.0-0.06)
AD_LO	0.0114 (0.0-0.03)	0.006 (0.0-0.02)	0.0059 (0.0-0.02)	0.6743 (0.66-0.69)	0.0145 (0.0-0.04)	0.007 (0.0-0.02)	0.0088 (0.0-0.03)	0.009 (0.0-0.02)	0.0113 (0.0-0.04)
AD_AN	0.0115 (0.0-0.03)	0.0059 (0.0-0.02)	0.006 (0.0-0.02)	0.0076 (0.0-0.02)	0.6811 (0.65-0.71)	0.0069 (0.0-0.02)	0.0088 (0.0-0.02)	0.0089 (0.0-0.02)	0.0114 (0.0-0.03)
AD_HV	0.0114 (0.0-0.03)	0.0058 (0.0-0.02)	0.0059 (0.0-0.02)	0.0078 (0.0-0.02)	0.0144 (0.0-0.04)	0.6736 (0.66-0.68)	0.0088 (0.0-0.03)	0.009 (0.0-0.03)	0.0116 (0.0-0.03)
AD_VI	0.0115 (0.0-0.03)	0.006 (0.0-0.02)	0.0059 (0.0-0.02)	0.0078 (0.0-0.02)	0.0143 (0.0-0.04)	0.0071 (0.0-0.02)	0.6757 (0.66-0.69)	0.0089 (0.0-0.03)	0.0115 (0.01-0.04)
LG_CO	0.0116 (0.0-0.03)	0.006 (0.0-0.02)	0.0063 (0.0-0.02)	0.0078 (0.0-0.02)	0.0146 (0.0-0.04)	0.007 (0.0-0.02)	0.0088 (0.0-0.02)	0.6756 (0.66-0.69)	0.0115 (0.0-0.03)
EG_AL	0.0114 (0.0-0.03)	0.006 (0.0-0.02)	0.0059 (0.0-0.02)	0.0077 (0.0-0.02)	0.0145 (0.01-0.04)	0.0069 (0.0-0.02)	0.0087 (0.0-0.03)	0.0089 (0.0-0.03)	0.6781 (0.66-0.70)

(b) Historical gene flow (*M*) with 95% confident intervals estimates for European lobster *Homarus gammarus* using Migrate-n. The population from the Ionian Sea was excluded from analysis due to the small sample size. Source populations = rows, sink populations = columns. Population codes and colours used as in Figure 4.

Pop ID	AD_VE	AD_UM	AD_PU	AD_LO	AD_AN	AD_HV	AD_VI	EG_AL	LG_CO
AD_VE	0	2 (0.65-2.95)	0.99 (0.37-2.03)	4.1 (3.71-5.18)	1 (0.73-2.27)	1.62 (0.83-2.39)	2.52 (1.63-3.41)	2.05 (1.27-2.79)	2 (1.44-3.51)
AD_UM	2.33 (1.11-3.71)	0	4.17 (3.93-5.57)	11.36 (9.61-14.53)	8.58 (7.01-10.24)	5.57 (4.31-6.53)	5.42 (3.44-7.08)	2.39 (1.67-3.03)	3.46 (2.28-4.61)
AD_PU	3.99 (3.17-4.73)	7.86 (6.57-10.65)	0	6.12 (5.01-7.68)	2.66 (2.01-3.27)	5.71 (4.31-6.75)	3 (2.5-5.121)	4.5 (3.58-5.84)	1 (0.59-2.64)
AD_LO	3.91 (2.61-5.18)	2.82 (1.51-4.04)	1.98 (1.07-2.33)	0	2.31 (1.89-4.25)	3.43 (1.44-3.78)	1.76 (0.68-2.78)	1.2 (0.01-2.55)	1.76 (1.45-3.37)
AD_AN	5.39 (3.85-6.73)	3.3 (2.21-4.13)	1.94 (1.27-2.59)	4.22 (3.25-4.99)	0	3.6 (2.75-4.43)	3.95 (2.83-4.93)	2.65 (1.29-3.43)	1.93 (1.23-2.63)
AD_HV	1.86 (1.11-2.45)	2.49 (1.81-3.15)	2.52 (1.01-3.37)	4.1 (2.31-6.07)	2.73 (1.99-3.43)	0	4.6 (2.45-6.49)	2.27 (1.43-3.17)	2.5 (2.21-4.21)
AD_VI	3.44 (2.35-4.23)	0.9 (0.73-2.05)	1.63 (0.57-2.43)	1.51 (0.67-2.27)	2.78 (1.64-3.91)	3.26 (2.45-4.03)	0	0.83 (0.5-2.08)	2.01 (1.29-2.65)
EG_AL	1.3 (0.97-2.05)	0.88 (0.21-1.53)	0.9 (0.51-1.69)	1.9 (1.23-2.53)	2.87 (2.07-3.69)	2.96 (1.97-3.27)	4.28 (3.44-5.81)	0	1.27 (0.81-3.75)
LG_CO	3.03 (1.98-3.85)	2.44 (1.65-3.19)	1.57 (0.77-2.39)	1.63 (1.19-3.33)	2.21 (1.57-2.85)	1.21 (0.59-1.81)	2.26 (0.58-3.11)	1.4 (0.59-2.19)	0

Supplementary Table S4. Distribution of effective population size *N*e per age class for both females and male individuals of European lobster *Homarus gammarus* sampled in the Adriatic Sea. AgeStructure (Wang et al., 2010) was used for *N*e estimation based on 159 males and 107 females.

	***N*e**		
**AgeClass**	**Females**	**Males**	**Δ**
1	78.9	63.9	15
2	78.9	63.9	15
3	78.9	63.9	15
4	78.9	60.6	18.3
5	76.6	59	17.6
6	74	57.5	16.5
7	72.5	56.2	16.3
8	70.1	55	15.1
9	67.8	53.4	14.4
10	65.5	51.9	13.6
11	63.4	50.3	13.1
12	61	49	12
13	58.6	46.9	11.7
14	57.8	45.9	11.9
15	57.4	44.2	13.2
16	54.5	40.4	14.1
17	16	12.5	3.5

Supplementary Table S5. Genetic diversity and neutrality tests (Tajima's D and Fu's FS) and mismatch distribution analysis under the sudden expansion model for the mitochondrial DNA COI sequences of European lobster *Homarus gammarus* collection from the Adriatic Sea. Population codes as in Table 1.

	Genetic diversity estimates				Neutrality tests		Spatial expansion parameters	
	**Haplotypes (H)**	**Polymorphic sites (S)**	**Haplotype diversity (Hd)**	**Nucleotide diversity (π)**	**Tajima's D (P) x10^{-3}**	**Fu's FS (P)**	**Tau**	**SSD (P)**
**Population**								
AD_IS	3	3	0.60 ± 0.13	1.97 ± 1.58	0.247 (0.693)	0.723 (0.621)	1.971	0.050 (0.311)
AD_LO	3	4	0.46 ± 0.20	1.74 ± 1.49	-1.535 (0.042)	0.204 (0.465)	2.777	0.031 (0.540)
AD_HV	5	7	0.62 ± 0.16	2.47 ± 1.84	-1.650 (0.038)	-1.204 (0.144)	3.155	0.011 (0.742)
AD_VI	4	6	0.65 ± 0.12	2.47 ± 1.80	-0.893 (0.205)	0.358 (0.556)	0.322	0.004 (0.802)
AD_BA	2	3	0.67± 0.31	3.48 ± 3.29	-	1.609 (0.674)	3.488	0.284 (0.193)
**Region**								
North_AD	5	6	0.44 ± 0.12	1.48 ± 1.21	-1.422 (0.061)	-1.304 (0.124)	1.880	0.005 (0.701)
Mid_AD	5	7	0.62 ± 0.10	2.39 ± 1.70	-0.802 (0.243)	-0.009 (0.509)	0.188	0.006 (0.734)
**Total**	6	7	0.46 ± 0.07	1.68 ± 1.29	-0.893 (0.229)	-0.918 (0.309)	1.804	0.001 (0.845)

Tau, expansion parameter; SSD, sum of squared deviations between observed and expected distributions; P-values are showed in parenthesis for different estimates.
