# Supplementary material for: Population Genetic Structure and Connectivity of the European Lobster Homarus gammarus in the Adriatic and Mediterranean Seas
Source: Front Genet. 2020 Dec 7;11:576023. doi: 10.3389/fgene.2020.576023 (PMC7750201; doi:10.3389/fgene.2020.576023)
Supplement: Supplementary file 3 [file Data_Sheet_3.pdf]

Supplementary Figures

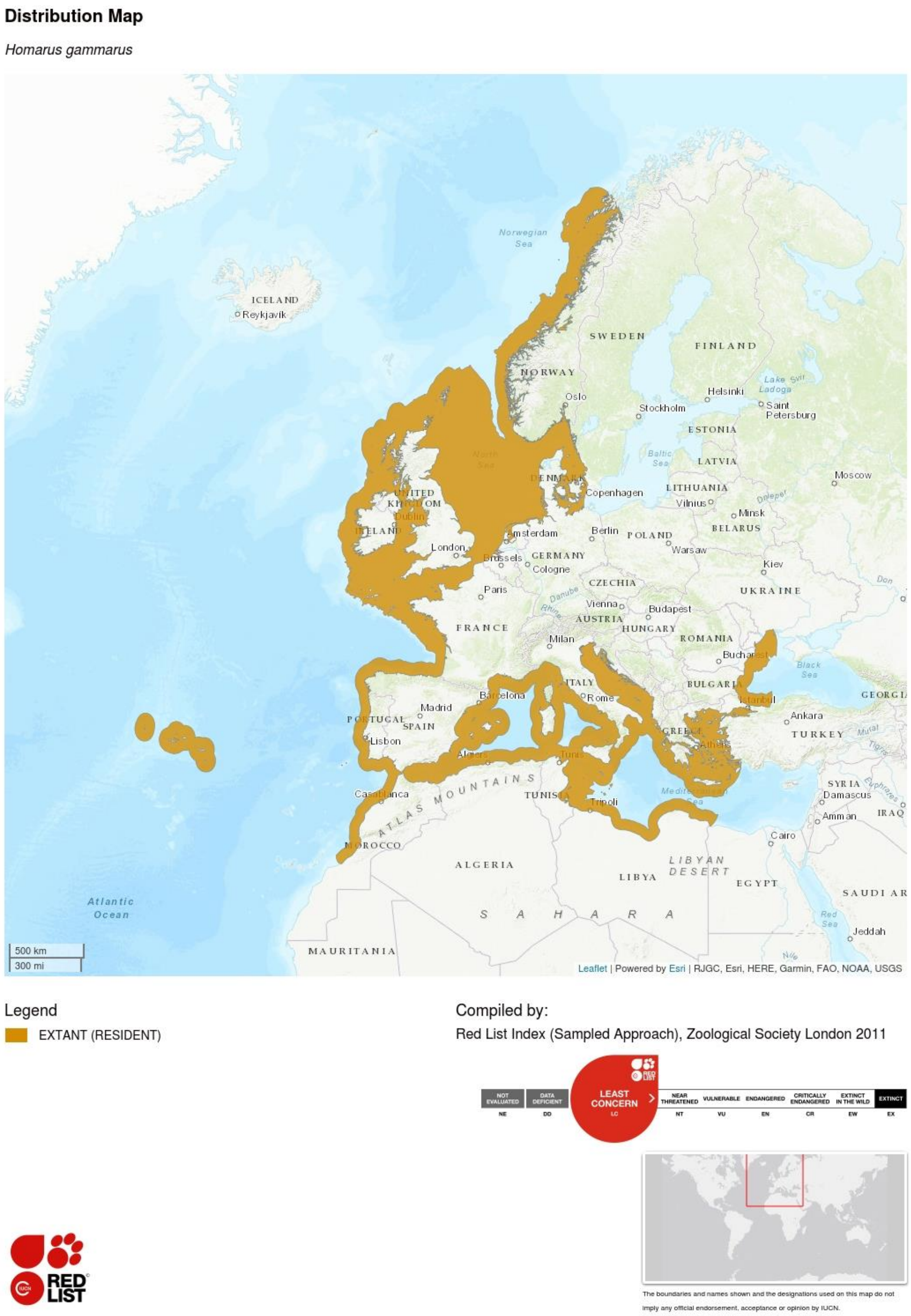

Supplementary Figure 1. Distribution map of the European lobster, *Homarus Gammarus* (source: https://www.iucnredlist.org/species/169955/69905303)

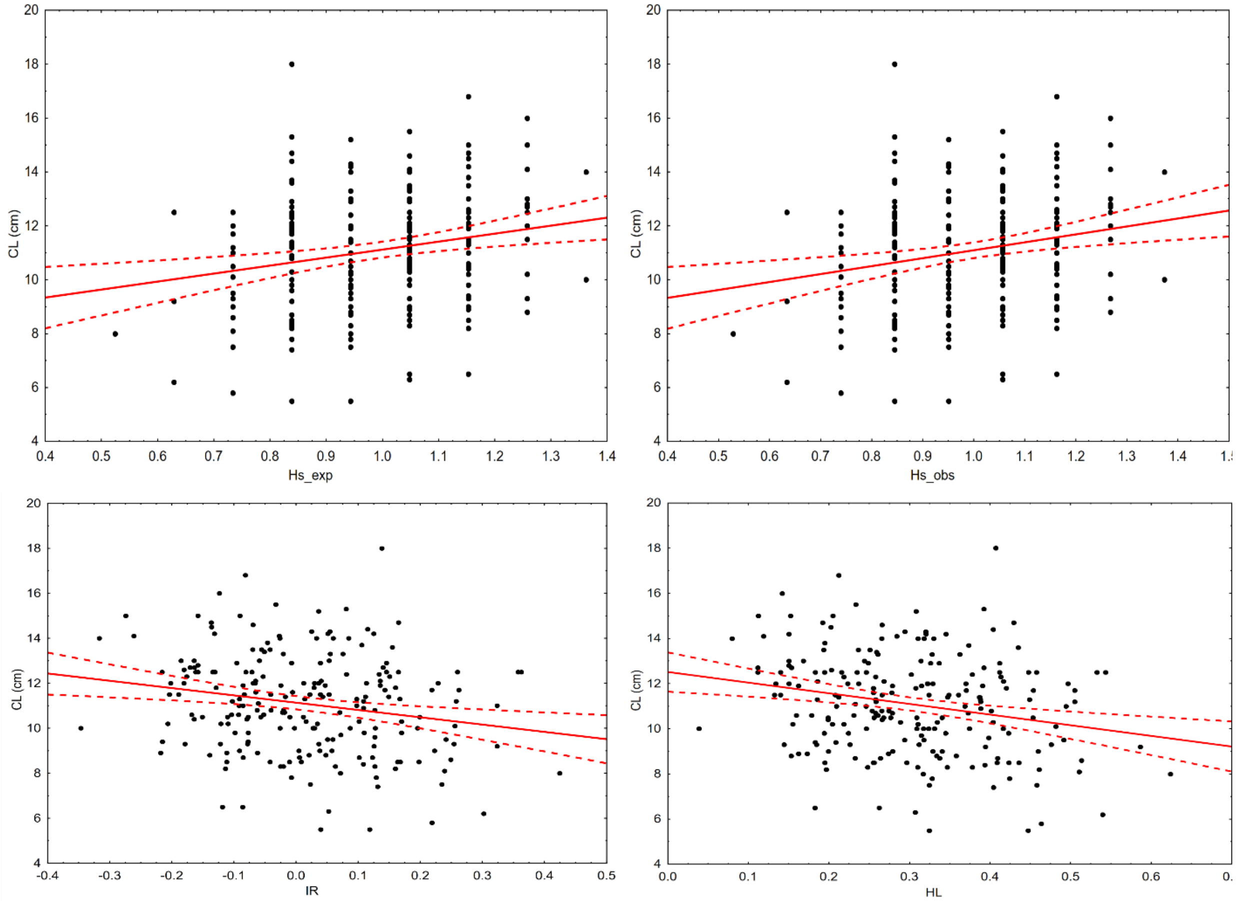

Supplementary Figure 2. Regression plots of the four different measures of heterozygosity, i.e. standardized observed heterozygosity per individual (Hs_exp, HS_obs), internal relatedness (IR) and homozygosity by locus (HL) in relation to carapace length in both male and female individuals.

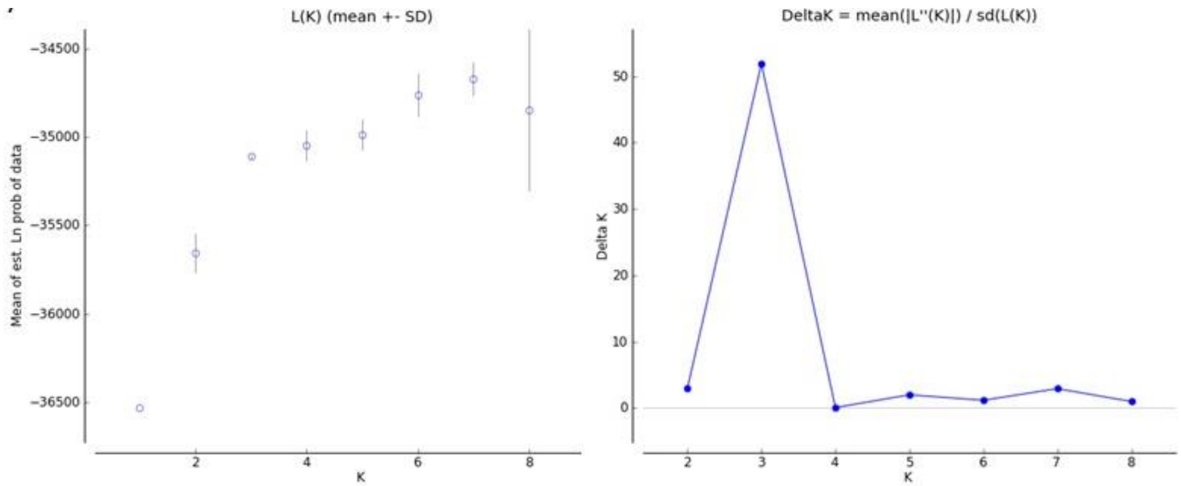

Supplementary Figure 3. Inference of the number of clusters in the European lobster *Homarus gammarus* population dataset. The first plot represents the values for the mean likelihood of each genetic cluster (K), where the error bar represents one standard deviation, while the second plot represents the values of Delta K calculated as the mean of the second-order rate of change in the likelihood of K divided by the standard deviation of the likelihood of K.

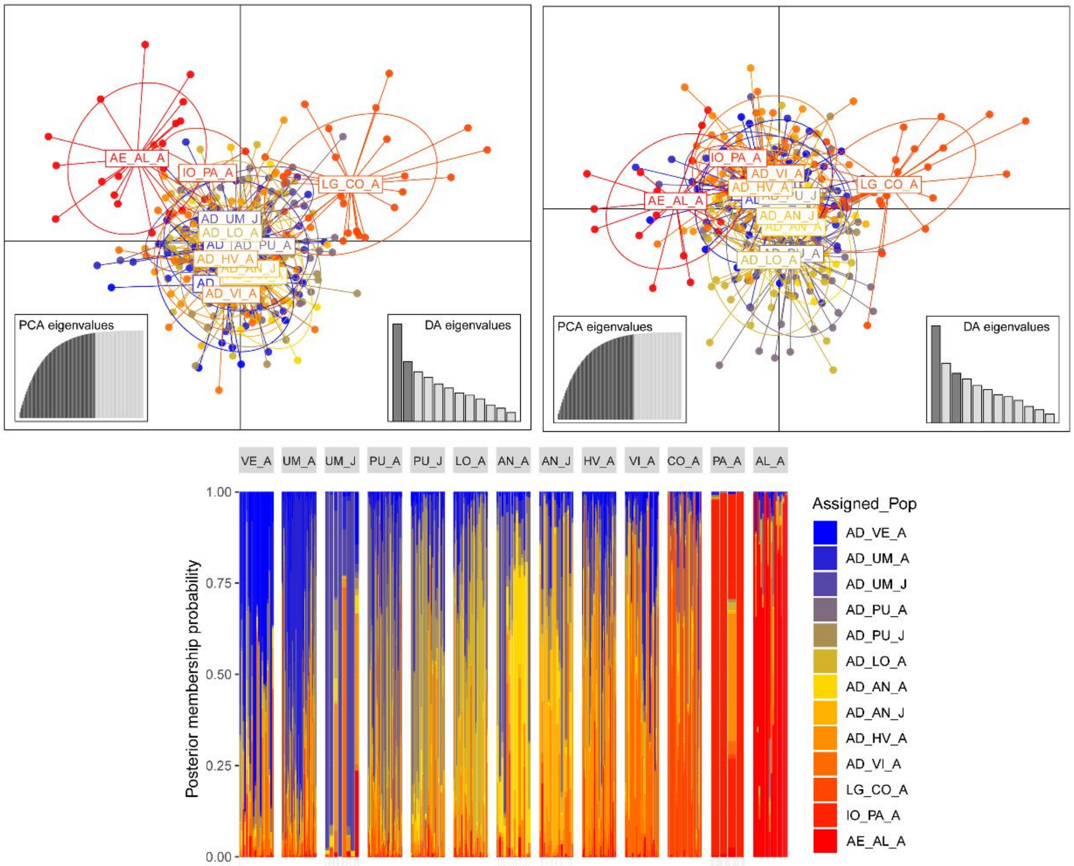

Supplementary Figure 4. Top: scatterplots of the final Discriminant Analysis of Principal Components (DAPC) model with sampling locality as a prior, where points are individuals, colour-coded by their original sampling locality and surrounded by a 95% confidence ellipse. DA and PCA Eigenvalues represent the amount of genetic variation captured by the analysis where the first scatter plot represents linear discriminant 1 vs. linear discriminate 2, and the second scatter plot represents linear discriminant 1 vs. linear discriminate 3. Bottom: membership plot of the posterior probability assignment of individuals in respect to their original populations. Populations codes are given in Table 1.

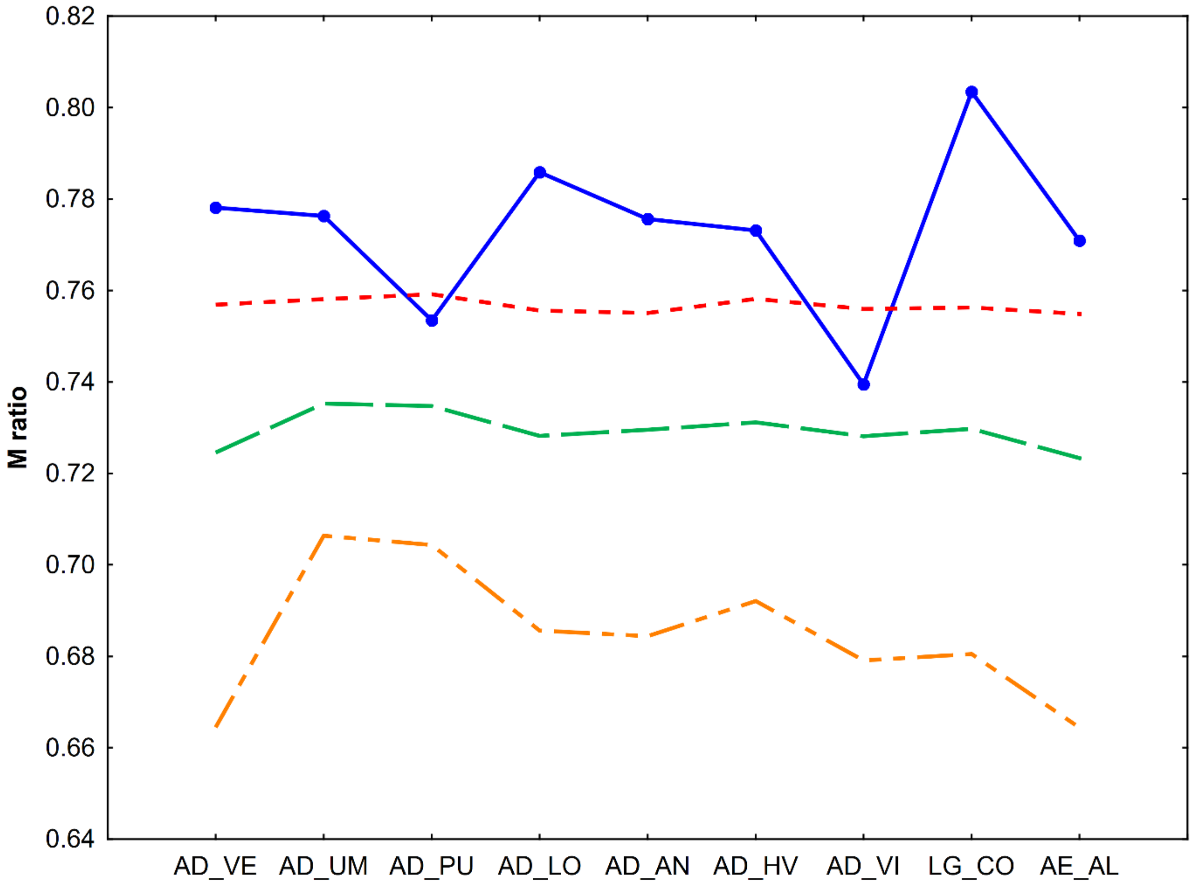

Supplementary Figure 5. Observed and simulated values of the Garza-Williamson index for European lobster *Homarus gammarus* populations. The observed *M*-ratio values are displayed as the solid blue line. The simulated critical *M* threshold (*M*c) values below which a bottleneck is evident was calculated for different ancestral theta and displayed as the short red dashed line ($\theta = 0.5$), long green dashed line ($\theta = 1$) and long/short orange dashed line ($\theta = 4$). Population *M*-ratio and *M*c parameters were calculated using M-P-Val and Critical M (Garza and Williamson, 2001). The population from the Ionian Sea was excluded from the analysis due to the small sample size. Populations codes are given in Table 1.
